# Classification of chinese fragrant rapeseed oil based on sensory evaluation and gas chromatography-olfactometry

**DOI:** 10.3389/fnut.2022.945144

**Published:** 2022-08-03

**Authors:** Fei Guo, Mingjuan Ma, Miao Yu, Qi Bian, Ju Hui, Xin Pan, Xiaoxia Su, Jihong Wu

**Affiliations:** ^1^College of Food Science and Nutritional Engineering, China Agricultural University, Beijing, China; ^2^COFCO Nutrition and Health Research Institute Co., Ltd., Beijing, China; ^3^Beijing Key Laboratory of Nutrition & Health and Food Safety, Beijing, China; ^4^Beijing Engineering Laboratory for Geriatric Nutrition Food Research, Beijing, China; ^5^National Engineering Research Center for Fruit and Vegetable Processing, Beijing, China; ^6^Key Laboratory of Fruit and Vegetable Processing, Ministry of Agriculture, Beijing, China; ^7^Beijing Key Laboratory for Food Non-thermal Processing, Beijing, China

**Keywords:** fragrant rapeseed oil, fragrance styles, sensory evaluation, gas chromatography-olfactometry, chemometrics methods

## Abstract

Fragrant rapeseed oils and traditional pressed oils are increasingly popular in China owing to their sensory advantages. Many fragrant rapeseed oils are labeled by different fragrance types; however, the scientific basis for these differences is lacking. To identify the distinctive aroma and achieve fragrance classification, the sensory characteristics and aroma components of nine different fragrant rapeseed oils were analyzed *via* sensory evaluation and gas-chromatography-mass spectrometry-olfactometry. A total of 35 aroma compounds were found to contribute to the overall aroma. By using chemometrics methods, rapeseed oils were categorized into three fragrance styles: “strong fragrance,” “umami fragrance,” and “delicate fragrance.” In total, 10 aroma compounds were predicted to be the most effective compounds for distinguishing sensory characteristics of fragrant rapeseed oil. According to our results, this approach has excellent potential for the fragrance classification and quality control of rapeseed oil.

## Introduction

Rapeseed, a traditional cash crop, is usually processed through roasting, screw pressing, and filtration to obtain edible oil in China. Fragrant rapeseed oil is a traditional pressing oil employed in China. According to the Chinese standard, pressing rapeseed oil is defined as a fragrant-pressing rapeseed oil that is prepared by roasting and squeezing rapeseed without the addition of other spices. The traditional processing of fragrant-pressing rapeseed oil is shown in Supplementary Figure S1. The consumption of fragrant rapeseed oil reached ~1.5 million tons (amounting to ~US 1.6 billion dollars) and has continued to increase (1). The aroma characteristics are the main driving factors of consumption, in terms of consumer preferences. Therefore, numerous products are being labeled with specific aroma advantages. However, a scientific basis is lacking to support these labels, ultimately creating chaos.

Sensory evaluation can be performed to quantify the intensity of a product's sensory characteristics. In fact, a sensory evaluation can effectively reflect the final aroma presented by a combination of aroma substances (2, 3), ultimately playing an essential role in fragrance classification (4, 5). Mao et al. explored changes in six flavor attributes of the rapeseed oil at different roasting temperatures using quantitative descriptive analysis (QDA) (6). As a result, the sensory evaluation results were found to always correlate with the aroma compound content of the rapeseed oil.

Several scholars have recently analyzed the aroma components of fragrant rapeseed oil. Wagner et al. identified 29 volatile components of virgin cold-pressed rapeseed oil during storage by gas-chromatography–mass spectrometry (GC–MS); however, all the components were not identified as aroma compounds (7). Zhou et al. identified six important aroma compounds in commercial rapeseed oil through gas chromatography–olfactometry (GC–O) (8). Of note, these studies have mainly focused on the volatile and aromatic compounds of rapeseed oils. Accordingly, there is a lack of systematic studies on fragrance classification, the identification of aroma components, and the correlation between sensory attributes and aroma compounds of fragrant rapeseed oil.

Chemometrics significantly contribute to the development of food sensory and aroma compounds. Correlation analysis between the parameters plays an important role in bridging the connection between the chemical and sensory data. Principal component analysis (PCA), hierarchical cluster analysis (HCA), and partial least squares regression (PLSR) have been successfully applied to explore the relationship between physical, chemical, and sensory data (9–11). According to the results of such studies, PLSR is confirmed as a highly effective tool for sensory quality control of mango (12) and commercial boletus (13).

In this study, nine fragrant rapeseed oils from the main production areas in China were selected as representative samples. The sensory characteristics of these oils were then evaluated using sensory analysis and the aroma compounds were identified using headspace solid–phase microextraction gas chromatography–mass spectrometry/olfactometry (HS–SPME–GC–MS/O). Based on the principal component analysis (PCA) and partial least squares regression (PLSR) analysis, the relationship between the sensory characteristics and aroma compounds was established, and the fragrance types of these different products were classified, which would be meaningful for quality control and the marketing strategies employed for industrial production.

## Materials and methods

### Fragrant rapeseed oils

In total, 9 samples were purchased from different rapeseed oil-producing areas in China, including the upper Yangtze River (Sichuan), lower Yangtze River (Jiangsu, Shanghai), and the plateau region (Yunnan) (details in Supplementary Table S4). In total, 9 samples of rapeseed oil are popular among local consumers. All the samples were produced using the traditional Chinese hot-pressing technology. Samples were stored at 4°C until further analysis.

### Chemicals

Acetaldehyde (95%), propanal (96%), 2-heptanone (97%), heptanal (98%), 2,5-dimethyl pyrazine (95%), 2,6-dimethyl pyrazine (95%), and 2-ethyl pyrazine (96%) were purchased from Sigma-Aldrich Co. Ltd (Shanghai, China). 5-Hepten-2-one, 6-methyl-(97%), dimethyl trisulfide (95%), 2-ethyl-3-methyl pyrazine (93%), 2-ethyl-6-methyl pyrazine (95%), furfural (97%), 2-ethyl-5-methyl pyrazine (98%), 2-ethyl-3,5-dimethyl pyrazine (96%), acetic acid (99%), benzaldehyde (97%), 1-butene, 4-isothiocyanato (94%), (E,E)-2,4-heptadienal (99%), (E)-2-nonenal (98%), dimethyl sulfoxide (97%), and 5-methyl-2-furancarboxaldehyde (97%) were supplied by CNW Technologies GmbH Co. Ltd (Shanghai, China). (E,Z)-2,6-Nonadienal (97%), butanoic acid (98%), (E)-2-decenal (99%), 2-furanmethanol (98%), (E,E)-2,4-nonadienal (99%), 2(5H)-furanone (97%), (E,E)-2,4-decadienal (96%), hexanoic acid (97%), benzyl nitrile (96%), heptanoic acid (99%), and benzenepropanenitrile (97%) were from Alfa Aesar reagent company (Shanghai, China).

### Sensory analysis

The sensory panel was composed of 10 experts with more than 2 years of experience in the sensory evaluation. The experts were recruited to comply with ISO standards and were selected based on their ability to identify and describe differences among oil samples (14). Quantitative descriptive analysis (QDA) was used to analyze the sensory characteristic intensity of fragrant rapeseed oils. The expert panel completed eight sessions in the sensory room. The first three sessions involved term generation based on the fragrant rapeseed oil samples. Subsequently, standardized evaluation skills were required. To formulate the scoring standards, the following sessions focused on the panelist training, including attribute learning, difference recognition, and intensity ranking. A proficiency test was then employed to check the evaluation ability of the experts to ensure the accuracy and consistency of the obtained data. The sensory attributes of fragrant rapeseed oil include roasted, pickle-like, burnt, green, pungent, and puffed food-like characteristics, the definitions of which are listed in Supplementary Table S5. The aforementioned six attributes were evaluated using a linear scale of 15 cm, where 0 indicates that the attribute was not detected and 15 indicates the strongest detection of a particular sensory characteristic. To ensure the consistency and repeatability of the evaluation results, blind samples were inserted into each test for verification. The oil samples were presented in random order during the sensory evaluation and served at 25°C in odorless cups with an effective volume of 10 ml. For each sensory evaluation test, the panelists assessed a maximum of five samples in separate compartments. Each panelist took a 1-min break between each sample to enable restoration of their sensory ability and prevent fatigue. The panelists drew a line on a 15-cm line scale, which indicated their perception of the sensory characteristics.

Shield light was applied during the complete sensory evaluation to alleviate the color interference of the testing samples. Boiled water and plain crackers were available for palate cleansing, and the final result was the average value of three replicates.

### Volatile compound analysis

According (HS–SPME–GC–MS) was used to measure the volatile compounds in the oil (8, 15). An oil sample of 5.00 ± 0.10 g and an internal standard material were added to a 20 ml vial, balanced at 60°C for 20 min, and extracted with a 2 cm 50/30 μm DVB/CAR/PDMS fiber (Supelco Ltd., Bellefonte, PA) for 40 min. After extraction, the fiber was desorbed in a split-less inlet at 250°C for 5 min.

An Agilent 7890B/5977A GC–MS instrument (Agilent Technologies Inc., Santa Clara, CA) was used for GC–MS analysis, with HP−5MS and DB–WAX chromatographic columns (30 m ×0.25 mm i.d., 0.25 μm film thickness, J&W Scientific, Folsom, CA). Helium was used as the carrier gas at a constant current of 1.20 ml/min. For the DB–WAX column, the internal standard, 2-octanol (50 μl; 0.819 mg/L, caprylic/capric triglyceride) was added. The initial oven temperature was 45°C, which was increased to 180°C at rate was 4°C /min; this temperature was held for 2 min, increased to 230°C at a rate of 10°C/min, and held for 6 min. For the HP−5MS column, the internal standard, 2-methyl-3-heptanone (10 μl; 0.816 mg/L, caprylic/capric triglyceride), was added. The initial oven temperature was 45°C, which was increased to 180°C at a rate of 4°C /min; this temperature was held for 5 min, increased to 250°C at a rate of 10°C /min, and held for 5 min. The electron energy of the MS was 70 eV, the temperature of the ion source was 250°C, and the scanning range was 40–500 *m/z*. By comparing their RI values relative to the C6–C23 n-alkanes, the volatile components were identified and obtained from the columns, retention times, standard substances, and NIST 2014 (National Institute of Standards and Technology, Gaithersburg, MD, USA). The relative content of each volatile component was calculated according to the normalized scanning total ion current peak area using the internal standard, and the final result was the average value of three replicates.

### Identification of the aroma compounds

The aromatic components in the oil samples were identified using an Agilent 7890B/5977A GC–MS coupled with a Sniffer (Sniffer 9000, Brechbuhler AG, Switzerland). The analysis conditions were the same as those employed for GC–MS. The DB–WAX column was employed, and the sniffing port was set at 230°C. In total, 5 well-trained panelists were selected for the GC–O analysis. In GC operation, the nose must be close to the sniffer port to record the aroma characteristics. Compounds identified by more than three panelists were selected as aroma compounds for further analysis.

### Relative odor activity value analysis

Relative odor activity value, which ranges from 0 to 100, was used to evaluate the contribution of the individual compounds to the entire aroma. To identify key odorous compounds in foods, the ROAV was calculated according to a method published by Zhang et al. (13). If the ROAV value of the aroma component was >1, the component could be considered the main contributor to the aroma of the sample (16). If the ROAV value was between 0.1 and 1, the component could be considered to have a specific effect on the overall aroma (17). Of note, the contributions of other compounds were considered minimal. The greater the ROAV value, the greater the aroma component that influences the overall aroma of the sample.

### Statistical analysis

Multivariate statistical analysis, including PCA, PLSR, and variable importance in projection (VIP) score analysis, was performed using the sensory and aroma component data on XLSTAT v.2016 (Microsoft Corporation, Redmond, Washington). Tukey's HSD *post-hoc* test (*p* < 0.05) was conducted to assess the statistical significance between aroma compounds *via* SPSS 22.0 statistical software (Chicago, Armonk, NY, USA). R 3.6.0 software (R Foundation for Statistical Computing, Vienna, Austria) was used to conduct Pearson correlation coefficient (*r*) analysis of the sensory data to identify correlations among the variables. The heatmap was visualized using Hemel (version 1.0, The Cuckoo Workgroup, Wuhan, China). Panel performance was monitored using Panel Check Software (Version 1.3.2, www.panelcheck.com).

## Results and discussion

### Sensory analysis

**Figure 1:**
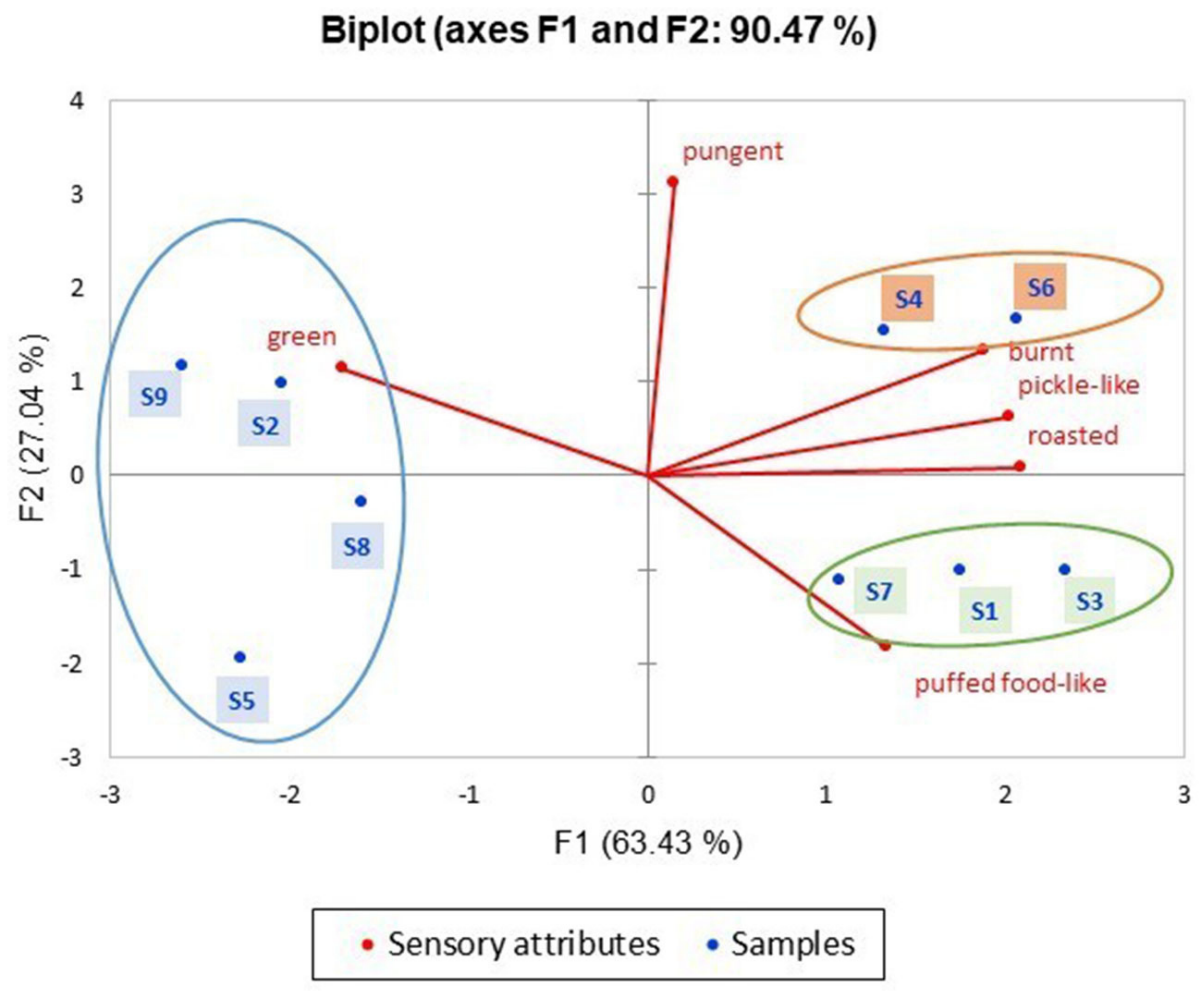
Principal component analysis diagrams of the nine fragrant rapeseed oils.

**Figure 2:**
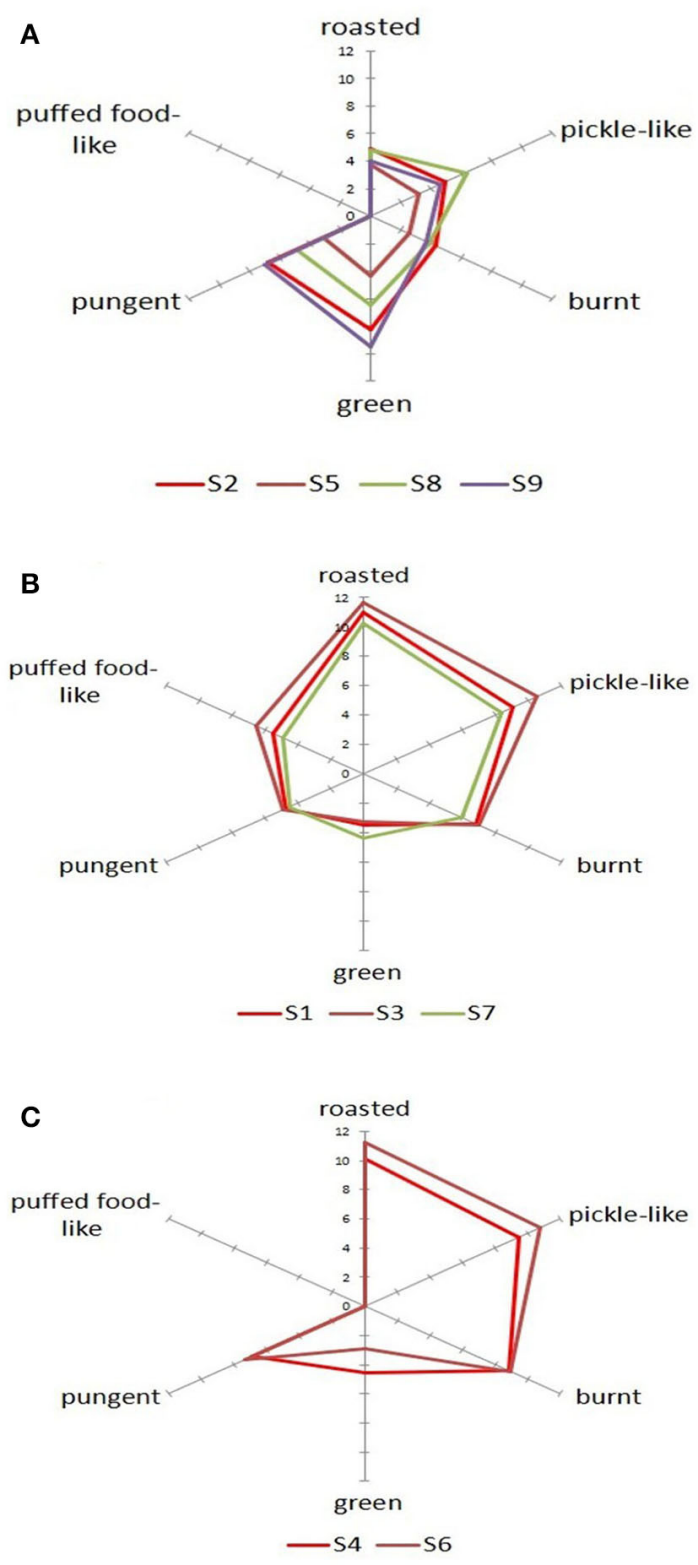
Distribution diagrams of the sensory characteristics of the nine fragrant rapeseed oils. **(A)** Delicate fragrance. **(B)** Umami fragrance. **(C)** Strong fragrance.

Quantitative descriptive analysis is a commonly applied descriptive sensory analysis for measuring the intensity of the sensory characteristics of fragrant rapeseed oil. By applying PCA to the sensory results of the nine samples to clarify their fragrance classification (Supplementary Table S1, Figure 1), the nine samples could be classified into three groups. Radar plots were used to illustrate the sensory characteristic distribution of each sample by determining the sensory characteristics of the three groups (Figure 2). The two figures show that the nine rapeseed oils had obvious differences in their fragrance styles. In total, 4 of the samples, namely, S2, S5, S8, and S9, were similar, with lower intensity of roast, pickled-like, and burnt odor, and higher intensity of the green odor. As a result, these samples were assigned to the “delicate fragrance” category. Samples S1, S3, and S7 were also similar, with a prominent puffed food-like structure, and were assigned to the “umami fragrance” category. The two remaining samples, S4 and S6, were similar as their four sensory characteristics of pickle-like, roasted, burnt, and pungent were relatively prominent. Accordingly, these samples were assigned to the “strong fragrance” category. Different fragrant rapeseed oils exhibit different sensory characteristics. Grass, nutty, roasted, and burnt were the main aromas from virgin rapeseed oil (18). The pungent attribute is the key characteristic used to differentiate between rapeseed oil samples (19).

To analyze the correlations between the sensory characteristics of fragrant rapeseed oil, the Pearson's correlation was used to measure the direct statistical relationship or association between two continuous variables (Supplementary Figure S2). Roasted food was found to be positively correlated with pickle-like (*r* = 0.95, *sig* < 0.0001) and burnt (*r* = 0.89, *sig* = 0.001) attributes, but negatively correlated with green (*r* = −0.77, *sig* = 0.014), and less correlated with pungent and puffed food-like. Pickle-like was positively correlated with burnt (*r* = 0.94, *sig* = 0.0002) and negatively correlated with green (*r* = −0.69, *sig* = 0.038) attributes. Therefore, strong positive correlations were found between roasted, burnt, and pickle-like attributes, which negatively correlated with green.

### Analysis of the volatile compounds

The volatile compounds in the nine fragrant rapeseed oils were identified by HS–SPME–GC–MS. A total of 158 volatile components were identified using the DB–WAX column and HP−5MS column, including 15 nitriles, 17 sulfides, 10 alcohols, 3 phenols, 31 N-heterocycles, 4 O-heterocycles, 30 aldehydes, 15 acids, 27 ketones, 2 olefins, and 4 esters (Supplementary Table S2). The volatile components in fragrant rapeseed oil are produced mainly through complex reactions, such as the Maillard reaction, glucosinolate (GLS) degradation, lipid peroxidation, and amino acid degradation (19). GLS degradation widely occurs in the metabolites of cruciferous plants, mainly producing sulfides and nitriles, which is the main reason for the pungent aroma of cold-pressed rapeseed oils (20). Differences in the nitriles and sulfides in the samples may be linked to the GLS composition and content of the different rapeseed varieties (6). The nitrile and sulfide contents of the “strong fragrance” rapeseed oil samples were higher than those of other samples, such as S4 (112.87 mg/kg) and S6 (108.89 mg/kg), but were little difference in the other two fragrance types. During seed roasting, GLS degradation tends to produce low-carbon nitriles and sulfide compounds, such as 4-isothiocyanato-1-butene and 2-isothiocyanato-butane (6). Among the samples, “strong fragrance” rapeseed oil had the highest amount of 4-isothiocyanato-1-butene. Some sulfide compounds can be further hydrolyzed to form low-molecular sulfides and disulfides (21). Low-molecular-weight sulfides and disulfides, such as carbon disulfide, dimethyl disulfide, dimethyl sulfoxide, and dimethyl trisulfide, were detected in nine fragrant rapeseed oils.

During the heating process in which the rapeseeds are roasted, many N-heterocycle and O-heterocycle compounds are produced by the Maillard reaction and amino acid degradation, such as pyrazine, pyridine, and furanone (22). Studies have shown that pyrazines are essential compounds in edible vegetable oils (15). A total of 16 pyrazines were identified in the nine fragrant rapeseed oils, with most of these compounds found in “umami fragrance” rapeseed oils (S1 and S7). 2,6-dimethylpyrazine was detected in all nine oils, which is similar to the results of Zhou, who identified two compounds in the commercial fragrant rapeseed oil (8). Aldehydes, alcohols, ketones, acids, and esters are mainly involved in the lipid oxidation, Strecker degradation, and amino acid degradation (23). Hexanal, (E)-2-octenal, and nonanal were identified in nine fragrant rapeseed oils. Ren et al. (24) also found hexanal and nonanal in the rapeseed oil. Of note, acetic acid had the highest content of all acids; however, its contents in the different types of fragrant rapeseed oils were found to markedly vary. Furaneol and 2,3-pentanedione were only found in the “umami fragrance” rapeseed oils (S1, S3, and S7) and have not been reported in the previous studies on rapeseed oils (6, 8, 24, 25).

### Analysis of the aroma compounds

The aroma compounds were identified by GC–O analysis. As shown in Table 1 and Supplementary Figure S3, 38 aroma compounds were detected in nine rapeseed oils, including two nitriles, four sulfides, two alcohols, one phenol, seven N-heterocycles, two O-heterocycles, 13 aldehydes, four acids, and three ketones. Tukey's HSD *post-hoc* test (*p* < 0.05) was employed to determine whether the 38 aroma compounds were significantly different between the rapeseed oils and could serve as critical markers for distinguishing between fragrant rapeseed oils (Table 1).

**Table 1:** Aroma compounds and their contents in fragrant rapeseed oils with significant differences (*p* < 0.05) analysis.

**No**.	**CAS**	**Compounds**	**Retention index**		**Aroma** **descriptors^1^**	**Concentration (mg/kg)** ^**2**^								
			**DB-WAX**	**HP-5MS**		**S1**	**S2**	**S3**	**S4**	**S5**	**S6**	**S7**	**S8**	**S9**
V1	74-93-1	methanethiol	694	464	Cabbage-like	0.14^a^	ND^e^	0.05^c^	ND^e^	ND^e^	0.04^d^	0.12^b^	ND^e^	ND^e^
V2	75-07-0	Acetaldehyde^3^	713	412	Green	0.37^c^	ND^f^	0.36^c^	0.42^b^	0.08^e^	0.32^d^	0.46^a^	0.08^e^	ND^f^
V3	123-38-6	Propanal^3^	801	506	Grass-like	ND^b^	ND^b^	ND^b^	ND^b^	ND^b^	ND^b^	ND^b^	ND^b^	0.58^a^
V4	96-17-3	2-methyl butanal	915	659	Cocoa-like	ND^e^	ND^e^	0.12^d^	0.14^b^	ND^e^	0.15^a^	0.13^c^	ND^e^	ND^e^
V5	600-14-6	2,3-Pentanedione	1,071	700	Nutty	0.08^a^	ND^d^	0.07^b^	ND^d^	ND^d^	ND^d^	0.04^c^	ND^d^	ND^d^
V6	624-92-0	dimethyl disulfide	1,072	740	Cabbage-like	0.08^d^	0.04^f^	0.06^e^	0.11^b^	0.02^h^	0.06^e^	0.09^c^	0.16^a^	0.03^g^
V7	110-43-0	2-heptanone^3^	1,187	900	Herbal	ND^d^	0.09^b^	ND^d^	ND^d^	0.03^c^	ND^d^	ND^d^	0.03^c^	0.17^a^
V8	111-71-7	Heptanal^3^	1,188	903	Green	ND^e^	0.24^b^	ND^e^	ND^e^	0.06^d^	ND^e^	ND^e^	0.16^c^	0.58^a^
V9	123-32-0	2,5-dimethyl pyrazine^3^	1,318	913	Cocoa-like	0.77^f^	1.00^e^	1.87^c^	6.47^a^	ND^g^	3.57^b^	0.78^f^	0.77^f^	1.23^d^
V10	108-50-9	2,6-dimethyl pyrazine^3^	1,325	912	Coffee-like	0.60^c^	0.18^e^	0.57^c^	0.93^b^	0.01^g^	1.47^a^	0.48^d^	0.11^f^	0.18^e^
V11	13925-00-3	2-ethyl pyrazine^3^	1,334	917	Nutty	0.54^c^	ND^d^	0.96^a^	ND^d^	ND^d^	ND^d^	0.74^b^	ND^d^	ND^d^
V12	110-93-0	5-hepten-2-one, 6-methyl-^3^	1,341	980	Green	ND^e^	0.95^c^	ND^e^	2.31^a^	0.46^d^	1.49^b^	ND^e^	0.80^c^	2.36^a^
V13	3658-80-8	dimethyl trisulfide^3^	1,383	963	Sulfur	ND^e^	0.04^c^	0.04^c^	ND^e^	ND^e^	0.01^d^	ND^e^	0.22^a^	0.07^b^
V14	15707-23-0	2-ethyl-3-methyl pyrazine^3^	1,397	999	Nutty	0.23^cd^	0.14^d^	0.30^c^	0.82^b^	ND^e^	0.95^a^	0.15^d^	0.12^d^	ND^e^
V15	13925-03-6	2-ethyl-6-methyl pyrazine^3^	1,402	992	Roasted potato	0.25^c^	0.20^d^	0.30^a^	ND^g^	ND^g^	ND^g^	0.16^e^	0.09^f^	0.27^b^
V16	13360-64-0	2-ethyl-5-methyl pyrazine^3^	1,415	1,001	Nutty	0.03^d^	0.16^c^	0.32^b^	0.84^a^	ND^d^	0.87^a^	ND^d^	0.13^c^	0.15^c^
V17	13925-07-0	2-ethyl-3,5-dimethyl pyrazine^3^	1,443	1,088	Nutty	ND^e^	0.37^d^	ND^e^	2.27^a^	ND^e^	ND^e^	ND^e^	0.48^c^	0.57^b^
V18	3386-97-8	1-butene, 4-isothiocyanato^3^	1,452	1,006	Pungent	0.75^f^	0.97^e^	2.41^c^	9.35^b^	ND^h^	10.40^a^	0.59^g^	0.66f^g^	2.11^d^
V19	98-01-1	furfural^3^	1,473	830	Baked bread	4.78^e^	2.97^f^	12.83^c^	16.09^b^	0.10^i^	23.86^a^	6.21^d^	0.87^h^	1.84^g^
V20	64-19-7	acetic acid^3^	1,480	600	Sour	14.50^b^	3.15^e^	12.74^c^	8.23^d^	0.71^f^	19.90^a^	14.76^b^	1.15^f^	3.23^e^
V21	4313-03-5	(*E,E*)-2,4-heptadienal^3^	1,494	1,007	Fatty	ND^e^	0.20^c^	0.17^c^	ND^e^	ND^e^	0.27^b^	ND^e^	0.11^d^	1.26^a^
V22	100-52-7	benzaldehyde^3^	1,534	921	Bitter	0.19^d^	0.19^d^	0.26^c^	0.56^a^	ND^f^	0.45^b^	0.14^d^	0.07^e^	0.41^b^
V23	18829-56-6	(*E*)-2-nonenal^3^	1,543	1,171	Green	ND^b^	ND^b^	ND^b^	ND^b^	ND^b^	ND^b^	ND^b^	ND^b^	0.42^a^
V24	67-68-5	dimethyl sulfoxide^3^	1,574	827	Garlic-like	0.49^e^	0.22^f^	0.86^c^	1.32^a^	ND^i^	1.09^b^	0.57^d^	0.06^h^	0.14^g^
V25	620-02-0	5-methyl-2-furancarboxaldehyde^3^	1,591	964	Spicy	2.79^d^	0.87^e^	4.71^b^	3.69^c^	0.04^f^	11.03^a^	2.72^d^	0.19^f^	0.66^e^
V26	557-48-2	(*E,Z*)-2,6-nonadienal^3^	1,595	1,156	Green	ND^b^	ND^b^	ND^b^	ND^b^	ND^b^	ND^b^	ND^b^	ND^b^	0.08^a^
V27	107-92-6	butanoic acid^3^	1,630	850	Cheese-like	ND^d^	ND^d^	ND^d^	ND^d^	0.06^c^	ND^d^	ND^c^	0.11^b^	0.59^a^
V28	3913-81-3	(*E*)-2-decenal^3^	1,630	1,234	Fatty	ND^e^	0.02^c^	ND^e^	ND^e^	ND^e^	ND^e^	0.01^d^	0.09^a^	0.08^b^
V29	98-00-0	2-furanmethanol^3^	1,678	864	Baked bread	1.43^a^	0.20^d^	0.36^c^	ND^e^	ND^e^	0.57^b^	0.30^c^	0.01^e^	0.04^e^
V30	5910-87-2	(*E,E*)-2,4-nonadienal^3^	1,704	1,204	Green	ND^b^	ND^b^	ND^b^	ND^b^	ND^b^	ND^b^	ND^b^	ND^b^	0.07^a^
V31	497-23-4	2(5H)-furanone^3^	1,767	915	Buttery	0.70^b^	0.09^e^	ND^g^	0.50^d^	ND^g^	0.88^a^	0.62^c^	0.03^f^	ND^g^
V32	2363-88-4	2,4-decadienal	1,767	1,284	Green	ND^b^	ND^b^	ND^b^	ND^b^	ND^b^	ND^b^	ND^b^	ND^b^	0.08^a^
V33	25152-84-5	(*E,E*)-2,4-decadienal^3^	1,826	1,326	Oily	ND^b^	ND^b^	ND^b^	ND^b^	ND^b^	ND^b^	ND^b^	ND^b^	0.28^a^
V34	142-62-1	hexanoic acid^3^	1,854	1,008	Sour	0.36^c^	0.17^de^	0.29^cd^	1.57^a^	0.10^e^	0.62^b^	0.24^cd^	0.16^de^	0.71^b^
V35	140-29-4	benzyl nitrile^3^	1,931	1,140	Pungent	ND^e^	0.02^d^	0.03^d^	0.17^b^	ND^e^	0.22^a^	ND^e^	ND^e^	0.08^c^
V36	111-14-8	heptanoic acid^3^	1,960	1,080	Sour	ND^d^	0.10^b^	ND^d^	ND^d^	ND^d^	ND^d^	0.06^c^	ND^d^	0.19^a^
V37	2785-89-9	4-ethyl-2-methoxy phenol	2,033	1,243	Smoky	ND^b^	ND^b^	ND^b^	ND^b^	ND^b^	ND^b^	ND^b^	ND^b^	0.08^a^
V38	645-59-0	benzenepropanenitrile^3^	2,048	1,244	Spicy	0.60^f^	0.84^e^	1.96^c^	6.34^b^	0.17^g^	7.05^a^	0.43^f^	1.16^d^	2.02^c^

“ND” Not detected.

1 Odor perceived at sniffing port.

2 Aroma compounds were identified by DB–Wax column. Values in the same row followed by the same letter were not significantly different by Tukey's HSD post-hoc testing (p < 0.05).

3 Identification using the authentic standards.

The ROAV is increasingly applied to evaluate the contribution of aroma compounds to the entire odor of samples (16, 17, 26). In this study, 35 aroma compounds contributing to the overall aroma of fragrant rapeseed oils (*ROAV* > 0.1) were selected for further analysis, as shown in Supplementary Table S3.

In total, 2 nitrile and 4 sulfide aroma compounds were identified from the GLS degradation products, including dimethyl disulfide (cabbage-like), dimethyl trisulfide (sulfur), 4-isothiocyanato-1-butene (pungent), dimethyl sulfoxide (garlic-like), benzyl nitrile (pungent), and benzenepropanenitrile (spicy). Dimethyl disulfide and dimethyl trisulfide were also detected in cold-pressed rapeseed oil (25). Zhou et al. discovered that benzyl nitrile provides pungency in the commercial rapeseed oils (8). Furthermore, 4-isothiocyanato-1-butene was reported to be the main contributor to the pungent aroma of rapeseed oils (24).

Heterocyclic compounds play an important role in roasted, baked, and nutty aroma (15). Pyrazine compounds are intermediate products of the Maillard reaction that have a nutty and roasted aroma. These compounds include 2,5-dimethyl pyrazine (cocoa-like), 2,6-dimethyl pyrazine (coffee-like), 2-ethyl pyrazine (nutty), 2-ethyl-3-methyl pyrazine (nutty), 2-ethyl-6-methyl pyrazine (roasted potato), 2-ethyl-5-methyl pyrazine (nutty), and 2-ethyl-3,5-dimethyl pyrazine (nutty). The “strong fragrance” and “umami fragrance” rapeseed oils had a higher content of pyrazine aroma compounds and a stronger roast intensity than the “delicate fragrance” rapeseed oils. S4, a “strong fragrance” rapeseed oil, had the highest content of pyrazine aroma compounds (11.33 mg/kg), S5, a “delicate fragrance” rapeseed oil, had the lowest content (0.01 mg/kg). According to Wei et al. (22), 2,5-dimethyl pyrazine exists in different varieties of rapeseed oils. Herein, 2-ethyl pyrazine was only detected in “umami fragrance” rapeseed oils (S1, S3, and S7), with concentrations of 0.54, 0.96, and 0.74 mg/kg, respectively.

Methanethiol (cabbage-like) was mainly found in the “umami flavor” oils, such as S1, S3, and S7. Methanethiol is produced *via* the degradation of sulfur amino acids, such as cysteine, methionine, and s-methylmethionine, in the Maillard reaction (27). Herein, 2-furanmethanol (baked bread) was not detected in S4 or S5. Furthermore, the highest content of 2-furanmethanol was found in S1 (1.43 mg/kg). Ren et al. (24) also found 2-furanmethanol in microwave-pretreated rapeseed oils.

The aldehyde compounds in rapeseed oil mainly provide green and tallow aromas, such as acetaldehyde (green), propanal (grass-like), heptanal (green), and (E)-2-nonenal (green). Zhou et al. (8) identified heptanal as an aroma-active compound in commercial rapeseed oil. (E,E)-2,4-heptadienal (fatty), (E)-2-decenal (fatty), and (E,E)-2,4-decadienal (oily) were found in S8, with relatively higher contents than those found in “delicate fragrance” rapeseed oil.

Only one phenolic aroma compound was found among the aroma components. This compound, 4-ethyl-2-methoxy phenol, provided a smoky aroma in S9. According to previous studies, 4-ethyl-2-methoxy phenol is prominent in the roasted mustard seeds and may be produced during the rapeseed roasting process (28).

### Correlation analysis of the sensory characteristics and aroma components

**Figure 3:**
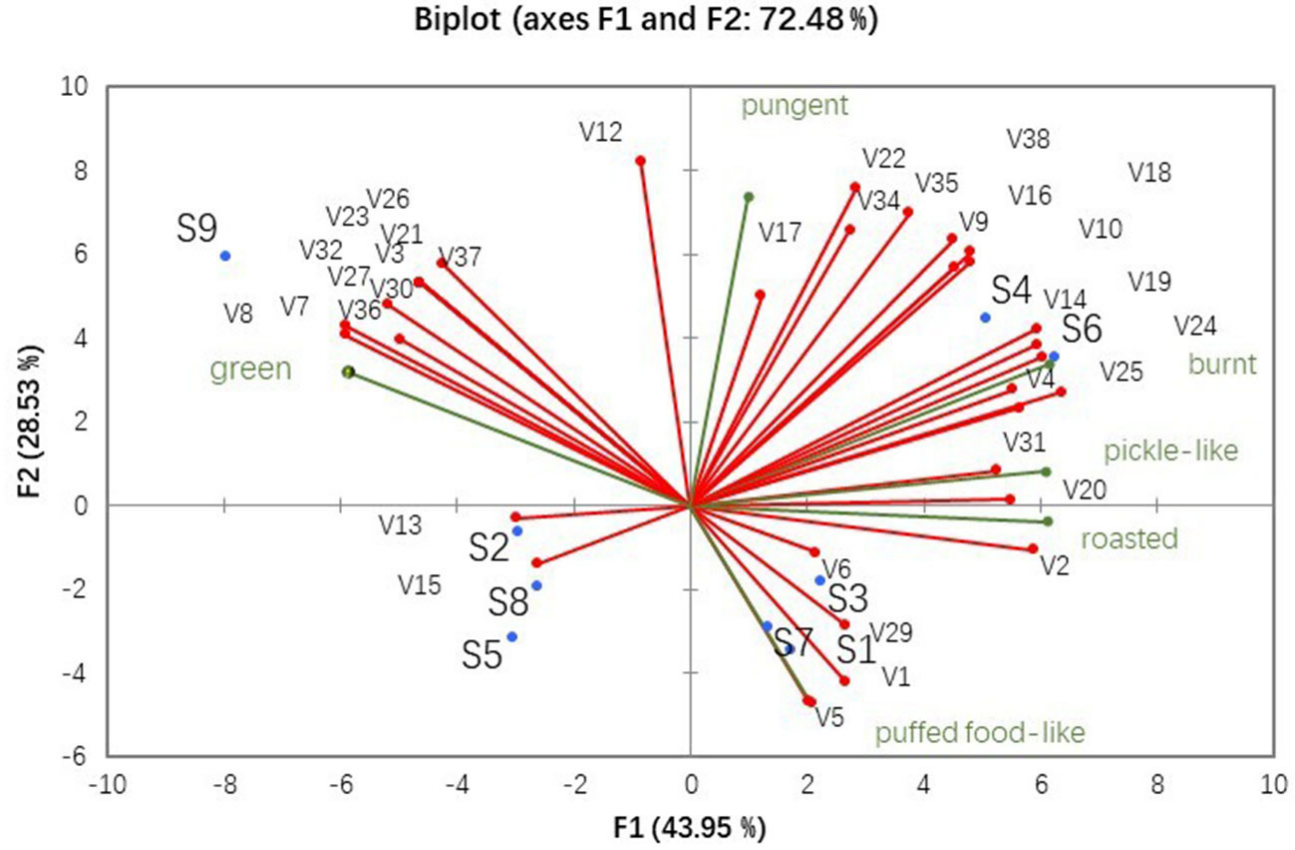
PCA analysis of nine fragrant rapeseed oils based on aroma compounds and sensory characteristics.

Principal component analysis was used to evaluate the correlation between the aroma compounds (Supplementary Table S3, *ROAV*>0.1) and sensory characteristics (Figure 2) of the nine fragrant rapeseed oils (Figure 3). The first two principal components accounted for 72.48% of the total variance, with the first principal component accounting for 43.95% and the second principal component accounting for 28.53% of the total.

As shown in Figure 3, S4 and S6 were located in the first quadrant, where roasted, burnt, and pickle-like sensory characteristics were surrounded by acetic acid (sour), 2(5H)-furanone (buttery), 5-methyl-2-furancarboxaldehyde (spicy), 2-methyl butanal (cocoa-like), dimethyl sulfoxide (garlic-like), furfural (baked bread), 2-ethyl-3-methyl pyrazine (nutty), 2-ethyl-5-methyl pyrazine (nutty), and 2,5-dimethyl pyrazine (cocoa-like). The left side of Figure 3 contains the S9, S2, S8, S5, and S6 samples (green). Few aroma compounds, such as 2-heptanone (herbal), heptanal (green), heptanoic acid (sour), butanoic acid (cheese-like), 2,4-decadienal (green), and (E,E)-2,4-nonadienal (green), were observed in this quadrant. Samples S1, S3, and S7 and puffed food-like were positioned in the fourth quadrant, which had dimethyl disulfide (cabbage-like), methanethiol (cabbage-like), 2,3-pentanedione (nutty), and 2-furanmethanol (baked bread). The pungent compound was positioned on the left side of the first quadrant. According to the Pearson's correlation analysis (Supplementary Figure S2) of the sensory characteristics, the pungent compound was correlated with burnt, roasted, pickle-like, and green, which may be produced by combining multiple compounds. PCA was also employed to identify the relationships between the nine fragrant rapeseed oils, which led to the same classification as the QDA. Notably, aroma compounds can explain the sensory characteristics of rapeseed oil.

**Figure 4:**
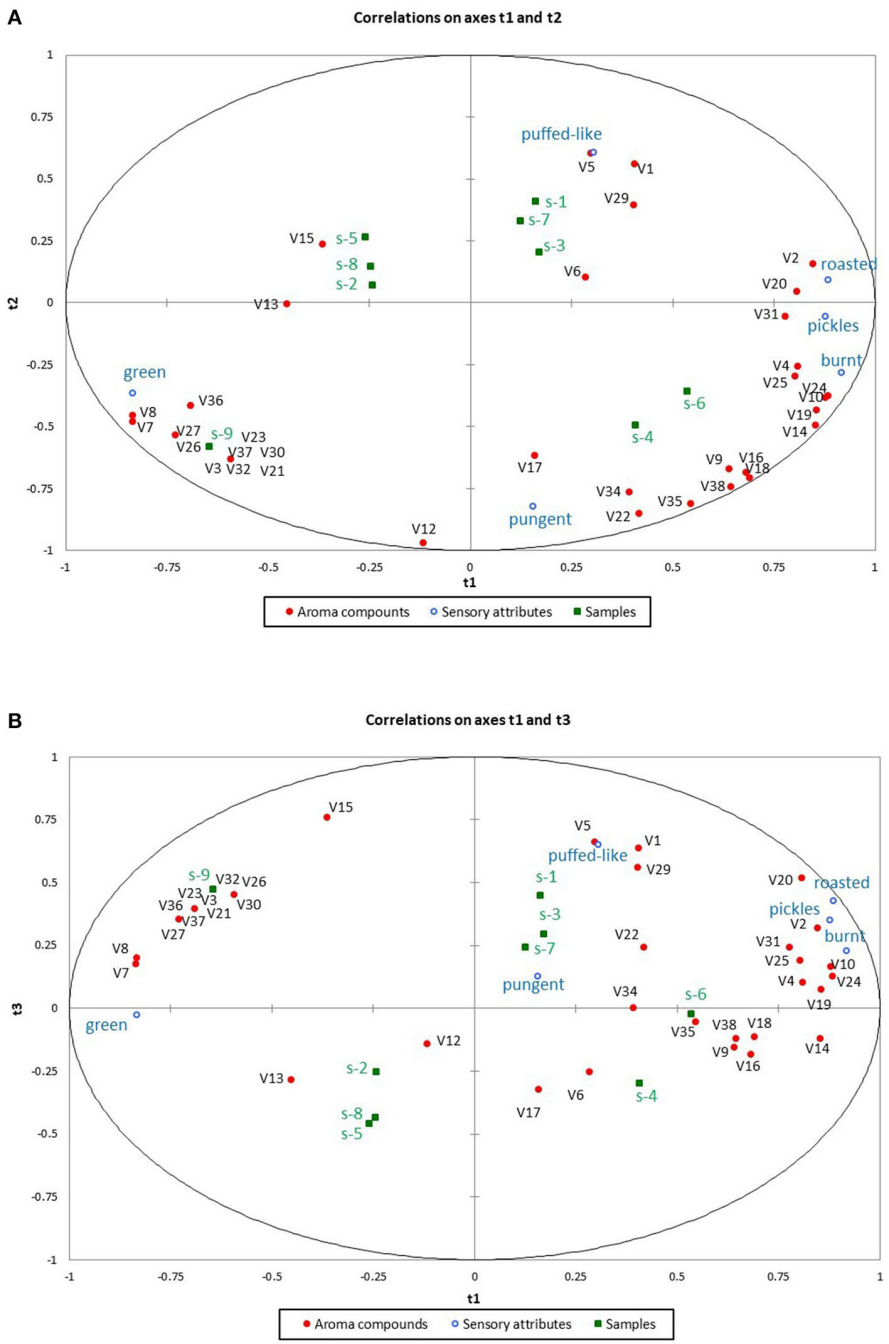
Partial least squares correlation model analysis of aroma compounds and sensory characteristics **(A)** axes t1 and t2. **(B)** axes t1 and t3.

To confirm the aforementioned findings, PLSR was used to determine the correlation between aroma compounds (Supplementary Table S3, *ROAV* > 0.1) and sensory characteristics (Figure 2). PLSR is a multivariate statistical analysis method that is applied to small sample sizes with many variables (29). The quality of the PLSR model was determined using cross-validation parameters. *R*^2^ and *Q*^2^ represent the variance and predictive capability, respectively. As shown in Figure 4, the *x* variables (*R*^2^*X* = 0.879) explain the variation in the *y* variables (*R*^2^*Y* = 0.841) according to the first three factors (*p* < 0.05, *Q*^2^ =0.701). The circle indicates a 100% explanation. Most variables were located around the circle, and no variables were collected in the center, indicating the reliability of the PLSR prediction model.

As shown in Figure 4, the umami fragrances of rapeseed oil samples (S1, S3, and S7) and puffed food-like were located on the upper right side of the chart. Meanwhile, 2, 3-pentanedione (nutty) and 2-ethyl pyrazine (nutty) were only detected in “umami fragrance” rapeseed oil samples. The “strong fragrance” rapeseed oil samples (S4 and S6) and the burnt, pickle-like and pungent aroma were located on the lower right side with many aroma components that promoted the burnt, pickle-like, and pungent aroma. The “delicate fragrance” rapeseed oil samples (S2, S5, S8, and S9) were located on the left side, close to the green aroma. Meanwhile, 2-heptanone (herbal) and heptanal (green) were only found in the “delicate fragrance” rapeseed oil samples. These results are consistent those of PCA and could be used to further categorize the nine rapeseed oils into three fragrance styles.

Aroma compounds with VIP values (*p* < 0.05) greater than one are considered the main reasons for the differences in the sensory characteristics. The VIP values for the 35 aroma compounds were > 1, including those of acetaldehyde (green), 2-methyl butanal (cocoa-like), 2-heptanone (herbal), heptanal (green), 2,6-dimethyl pyrazine (coffee-like), 2-ethyl-3-methyl pyrazine (nutty), furfural (baked bread), acetic acid (sour), dimethyl sulfoxide (garlic), and 5-methyl-2-furancarboxaldehyde (spicy). The SD was low (<0.35) and the results were statistically significant (*p* < 0.05). Thus, the 10 aroma compounds were significantly correlated with roasted, burnt, pickle-like, green, and puffed food-like compounds, which can be considered the main contributors for distinguishing the sensory characteristics of the nine fragrant rapeseed oils. A heat map was used to visualize the relationship between the nine fragrant rapeseed oil samples from the three fragrance styles and the 10 aroma compounds to derive the correlation, as shown in Supplementary Figure S4. Our findings indicate that the nine oils can be classified into three groups. Thus, the 10 aroma compounds play a vital role in the sensory characteristics of fragrant rapeseed oil.

## Conclusion

In conclusion, the findings of the present study revealed that roasted, pickle-like, burnt, green, pungent, and puffed food-like are the sensory characteristics of the fragrant rapeseed oil. Furthermore, 10 aroma compounds can be considered to be mainly responsible for the differences in the sensory characteristics. On the basis of the sensory and aroma compound analyses, the fragrant rapeseed oil can be classified into three different fragrance styles: “strong fragrance,” “umami fragrance,” and “delicate fragrance.” Our findings indicate that this approach has potential for application in the flavor classification and sensory quality control of fragrant rapeseed oil and could be used to establish an important basis for the market positioning, fragrance grading, and process improvement of fragrant edible oils in the future.

## Data availability statement

The original contributions presented in the study are included in the article/Supplementary materials, further inquiries can be directed to the corresponding authors.

## Author contributions

FG, XS, and JW designed the study. FG, MM, MY, and XP collected the data and participated in the design of the experimental. FG lead the data analysis, with the participation of QB and JH. All authors participated in manuscript preparation and revised the final version of the manuscript. All authors contributed to the article and approved the submitted version.

## Funding

This work was supported by the National Key Research and Development Program, China (Grant Number: 2017YFD0400106).

## Conflict of interest

The authors declare that the research was conducted in the absence of any commercial or financial relationships that could be construed as a potential conflict of interest.

## Publisher's note

All claims expressed in this article are solely those of the authors and do not necessarily represent those of their affiliated organizations, or those of the publisher, the editors and the reviewers. Any product that may be evaluated in this article, or claim that may be made by its manufacturer, is not guaranteed or endorsed by the publisher.

## References

[1] Zhang Y Zhu Y Shi L Guo Y Wei L Zhang H. Physicochemical properties and health risk assessment of polycyclic aromatic hydrocarbons of fragrant rapeseed oils in China. J Sci Food Agric. (2020) 100:3351–9. 10.1002/jsfa.10368 32162691

[2] Escuderos ME Uceda M Sánchez S Jiménez A. Instrumental technique evolution for olive oil sensory analysis. Eur J Lipid Sci Technol. (2007) 109:536–46. 10.1002/ejlt.200600239

[3] Lukić I Horvat L Godena S Krapac M Lukić M Vrhovsek U. Towards understanding the varietal typicity of virgin olive oil by correlating sensory and compositional analysis data: a case study. Food Res Int. (2018) 112:78–89. 10.1016/j.foodres.2018.06.022 30131161

[4] De Santis D Frangipane MT. Sensory perceptions of virgin olive oil: new panel evaluation method and the chemical compounds responsible. Nat Sci. (2015) 7:132. 10.4236/ns.2015.73015

[5] Borràs E Ferré J Boqué R Mestres M Aceña L Calvo A. Prediction of olive oil sensory descriptors using instrumental data fusion and partial least squares (PLS) regression. Talanta. (2016) 155:116–23. 10.1016/j.talanta.2016.04.040 27216664

[6] Mao X Zhao X Huyan Z Liu T Yu X. Relationship of glucosinolate thermal degradation and roasted rapeseed oil volatile odor. J Agric Food Chem. (2019) 67:11187–97. 10.1021/acs.jafc.9b04952 31552744

[7] Wagner C Bonte A Brühl L Niehaus K Bednarz H Matthäus B. Micro-organisms growing on rapeseed during storage affect the profile of volatile compounds of virgin rapeseed oil. J Sci Food Agric. (2018) 98:2147–55. 10.1002/jsfa.8699 28960362

[8] Zhou Q Xiao J Yao Y Wang B Wei C Zhang M. Characterization of the aroma-active compounds in commercial fragrant rapeseed oils via monolithic material sorptive extraction. J Agric Food Chem. (2019) 67:11454–63. 10.1021/acs.jafc.9b05691 31529950

[9] Cheng K Peng B Yuan F. Volatile composition of eight blueberry cultivars and their relationship with sensory attributes. Flavour Fragr J. (2020) 35:443–53. 10.1002/ffj.3583

[10] Pu D Zhang Y Sun B Ren F Zhang HF Chen H. Characterization of the key taste compounds during bread oral processing by instrumental analysis and dynamic sensory evaluation. LWT. (2021) 138:110641. 10.1016/j.lwt.2020.11064131285010

[11] Zielinski AA Haminiuk CW Nunes CA Schnitzler E Ruth SM Granato D. Chemical composition, sensory properties, provenance, and bioactivity of fruit juices as assessed by chemometrics: a critical review and guideline. Comprehens Rev Food Sci Food Safety. (2014) 13:300–16. 10.1111/1541-4337.12060 33412653

[12] Sung J Suh JH Chambers AH Crane J Wang Y. Relationship between sensory attributes and chemical composition of different mango cultivars. J Agric Food Chem. (2019) 67:5177–88. 10.1021/acs.jafc.9b01018 30977646

[13] Zhang H Huang D Pu D Zhang Y Chen H Sun B. Multivariate relationships among sensory attributes and volatile components in commercial dry porcini mushrooms (*Boletus edulis*). Food Res Int. (2020) 133:109112. 10.1016/j.foodres.2020.109112 32466923

[14] ISO. International Standard 8586. Sensory Analysis—General Guidelines for the Selection, Training and Monitoring of Selected Assessors and Expert Sensory Assessors (2014).

[15] Diez-Simon C Mumm R Hall RD. Mass spectrometry-based metabolomics of volatiles as a new tool for understanding aroma and flavour chemistry in processed food products. Metabolomics. (2019) 15:1–20. 10.1007/s11306-019-1493-6 30868334 PMC6476848

[16] Wei J Zhang Y Wang Y Ju H Niu C Song Z. Assessment of chemical composition and sensorial properties of ciders fermented with different non-Saccharomyces yeasts in pure and mixed fermentations. Int J Food Microbiol. (2020) 318:108471. 10.1016/j.ijfoodmicro.2019.108471 31841786

[17] Fan Y Liu W Xu F Huang Y Zhang H. Comparative flavor analysis of eight varieties of Xinjiang flatbreads from the Xinjiang Region of China. Cereal Chem. (2019) 96:1022–35. 10.1002/cche.10207

[18] Jing B Guo R Wang M Zhang L Yu X. Influence of seed roasting on the quality of glucosinolate content and flavor in virgin rapeseed oil. LWT. (2020) 126:109301. 10.1016/j.lwt.2020.109301

[19] Zhou Q Tang H Jia X Zheng C Huang F Zhang M. Distribution of glucosinolate and pungent odors in rapeseed oils from raw and microwaved seeds. Int J Food Properties. (2018) 21:2296–308. 10.1080/10942912.2018.1514632

[20] Kushad MM Brown AF Kurilich AC Juvik JA Klein BP Wallig MA. Variation of glucosinolates in vegetable crops of Brassica o leracea. J Agric Food Chem. (1999) 47:1541–8. 10.1021/jf980985s 10564014

[21] Pechacek R Velíšek J Hrabcová H. Decomposition products of allyl isothiocyanate in aqueous solutions. J Agric Food Chem. (1997) 45:4584–8. 10.1021/jf970316z10956150

[22] Wei F Yang M Zhou Q Zheng C Peng J Liu C. Varietal and processing effects on the volatile profile of rapeseed oils. LWT Food Sci Technol. (2012) 48:323–9. 10.1016/j.lwt.2012.04.007

[23] Xu L Yu X Li M Chen J Wang X. Monitoring oxidative stability and changes in key volatile compounds in edible oils during ambient storage through HS-SPME/GC–MS. Int J Food Properties. (2018) 20:1–13. 10.1080/10942912.2017.1382510

[24] Ren X Wang L Xu B Wei B Liu Y Zhou C. Influence of microwave pretreatment on the flavor attributes and oxidative stability of cold-pressed rapeseed oil. Drying Technol. (2019) 37:397–408. 10.1080/07373937.2018.1459682

[25] Zhou Q Mei Y Huang F Zhang C Deng Q. Effect of pretreatment with dehulling and microwaving on the flavor characteristics of cold-pressed rapeseed oil by GC-MS-PCA and electronic nose discrimination. J Food Sci. (2013) 78:C961–70. 10.1111/1750-3841.12161 23865448

[26] Wang M Zhang J Chen J Jing B Zhang L Yu X. Characterization of differences in flavor in virgin rapeseed oils by using gas chromatography–mass spectrometry, electronic nose, and sensory analysis. Eur J Lipid Sci Technol. (2020) 122:1900205. 10.1002/ejlt.201900205

[27] Zhang Y Liu Y Yang W Huang J Liu Y Huang M. Characterization of potent aroma compounds in preserved egg yolk by gas chromatography–olfactometry, quantitative measurements, and odor activity value. J Agric Food Chem. (2018) 66:6132–41. 10.1021/acs.jafc.8b01378 29790747

[28] Ortner E Granvogl M Schieberle M. Elucidation of thermally induced changes in key odorants of white mustard seeds (*Sinapis alba* L.) and rapeseeds (*Brassica napus* L.) using molecular sensory science. J Agric Food Chem. (2016) 64:8179–90. 10.1021/acs.jafc.6b03625 27690424

[29] Mehmood T Liland KH Snipen L Sæbø S. A review of variable selection methods in partial least squares regression. Chemom Intell Lab Syst. (2012) 118:62–9. 10.1016/j.chemolab.2012.07.01032342803

