# Supplementary material for: Classification of chinese fragrant rapeseed oil based on sensory evaluation and gas chromatography-olfactometry
Source: Front Nutr. 2022 Aug 3;9:945144. doi: 10.3389/fnut.2022.945144 (PMC9381969; doi:10.3389/fnut.2022.945144)

Table S1 Sensory characteristics intensity of fragrant rapeseed oils

	Roasted	Pickle-like	Burnt	Green	Pungent	Puffed food-like
S1	11.0 b	9.0 b	6.9 b	3.5 e	4.8 b	5.5 b
S2	4.9 d	4.9 e	4.3 d	8.2 b	6.8 a	0.0 d
S3	11.7 a	10.5 a	7.0 b	3.3 ef	4.9 b	6.6 a
S4	10.2 c	9.5 b	8.9 a	4.5 d	6.9 a	0.0 d
S5	3.8 e	3.1 f	2.6 f	4.3 d	3.1 c	0.0 d
S6	11.2 ab	10.8 a	8.9 a	2.9 f	7.3 a	0.0 d
S7	10.3 c	8.4 c	5.9 c	4.3 d	4.5 b	4.9 c
S8	4.8 d	6.3 d	3.8 e	6.5 c	4.9 b	0.0 d
S9	4.0 e	4.6 e	3.7 e	9.5 a	7.0 a	0.0 d

Note: Values in the same column followed by the same letter were not found to be significantly different by LSD post-hoc testing ($p<0.05$).

Table S2 Volatile compounds and their contents in fragrant rapeseed oils

NO.	Name	S1 (HP-5MS)	S1 (DB-WAX)	S2 (HP-5MS)	S2 (DB-WAX)	S3 (HP-5MS)	S3 (DB-WAX)	S4 (HP-5MS)	S4 (DB-WAX)	S5 (HP-5MS)	S5 (DB-WAX)
	**Nitriles**										
1	3-butenenitrile	-	0.18±0.00	0.3±0.01	0.61±0.00	1.04±0.01	3.27±0.06	2.62±0.05	2.71±0.01	-	-
2	methylacrylonitrile	-	0.19±0.00	0.04±0.00	-	-	-	-	-	-	-
3	2-butenenitrile	0.11±0.00	0.3±0.02	0.04±0.00	0.54±0.01	0.66±0.1	1.84±0.13	1.45±0.33	2.65±0.00	-	0.01±0.00
4	2,4-pentadienenitrile	0.21±0.00	0.84±0.07	0.04±0.00	0.17±0.00	0.23±0.04	0.82±0.01	0.31±0.03	1.11±0.1	-	0.01±0.00
5	(ch3)2c=chcn	3.87±0.12	13.39±0.23	4.08±0.05	19±0.24	10.76±0.11	26.71±0.09	39.27±0.7	57.69±0.23	0.35±0.01	1.99±0.02
6	5-cyano-1-pentene	1.74±0.13	4.23±0.06	1.71±0.03	9.89±0.16	4.41±0.05	8.44±0.06	17.76±0.16	22.42±0.23	0.13±0.01	0.43±0.02
7	2-methyl-butanenitrile	-	-	-	-	-	0.08±0.01	-	-	-	-
8	5-methyl-hexanenitrile	0.07±0.00	0.24±0.00	-	0.42±0.00	0.18±0.00	0.46±0.00	0.58±0.00	-	-	0.01±0.00
9	isoamyl cyanide	-	-	-	0.52±0.00	-	-	-	-	0.01±0.00	-
10	heptanonitrile	-	0.11±0.01	0.08±0.01	0.29±0.01	-	-	-	1.16±0.17	-	-
11	3-pentenenitrile	-	-	-	-	-	-	-	-	-	-
12	2-pentenenitrile	-	-	-	-	-	0.08±0.00	-	-	-	-
13	hexanenitrile	-	-	-	-	-	-	-	-	-	-
14	benzylnitrile	-	-	-	0.02±0.00	-	0.03±0.00	-	0.17±0.01	-	-
15	benzenepropanenitrile	1.02±0.01	0.6±0.00	1.03±0.04	0.84±0.13	3.87±0.31	1.96±0.08	15.3±0.62	6.34±0.28	0.07±0.00	0.17±0.01
	**Sulfides**										
16	thiazole	-	0.06±0.00	-	-	-	-	-	-	-	-
17	5-thiazoleethanol,4-methyl-	-	-	-	-	-	-	-	0.03±0.00	-	-
18	carbon disulfide	1.68±0.09	3.1±0.05	0.33±0.05	1.14±0.02	1.77±0.16	2.56±0.06	5.13±0.08	5.89±0.3	-	-
19	dimethyl disulfide	-	0.08±0.00	-	0.04±0.00	0.02±0.00	0.06±0.00	-	0.11±0.01	-	0.02±0.00
20	dimethyl sulfoxide	0.2±0.01	0.49±0.05	-	0.22±0.00	0.05±0.01	0.86±0.01	0.03±0.01	1.32±0.02	-	-

21	2(3h)-thiophenone,dihydro-5-methyl-	0.18±0.00	-	0.11±0.01	0.22±0.01	0.19±0.01	-	1.12±0.1	1.92±0.02	-	-
22	dimethyl trisulfide	-	-	-	0.04±0.00	-	0.04±0.00	-	-	-	-
23	cyano-3,4-epithiobutane	-	-	-	-	-	-	0.15±0.01	-	-	-
24	5-ethyl-4-methylthiazole	-	-	-	-	-	-	0.05±0.01	-	-	-
25	pentanenitrile,5-(methylthio)-	-	0.09±0.00	0.04±0.01	-	-	0.09±0.00	-	-	-	-
26	6-(methylthio)hexanenitrile	-	-	-	-	-	0.46±0.02	-	-	-	-
27	methanethiol	-	0.14±0.01	-	-	-	0.05±0.00	-	-	-	-
28	2-thiophenemethanol	-	-	0.02±0.00	-	0.13±0.02	0.08±0.00	-	-	-	-
29	3-thiophenecarboxaldehyde	-	0.23±0.01	-	-	-	0.22±0.00	-	-	-	-
30	butane,2-isothiocyanato	-	-	-	-	-	-	0.19±0.00	-	-	-
31	1-butene,4-isothiocyanato	-	0.75±0.00	0.69±0.01	0.97±0.02	2.19±0.12	2.41±0.04	10.44±0.39	9.35±0.19	-	-
32	benzene,(2-isothiocyanatoethyl)-	-	-	-	-	-	-	0.1±0.01	-	-	-
	**N-heterocycles**										
33	1,3-diazine	0.06±0.01	-	-	-	-	0.04±0.00	-	-	-	-
34	pyrazine	-	0.14±0.00	-	-	-	-	-	-	-	-
35	pyridine	0.18±0.01	0.69±0.00	0.02±0.00	-	0.05±0.00	-	-	-	-	-
36	3-methyl pyridine	0.02±0.00	-	-	-	0.01±0.00	-	-	-	-	-
37	methyl pyrazine	0.71±0.03	2.51±0.00	0.12±0.02	-	0.44±0.03	-	1.15±0.08	-	-	-
38	2,5-dimethyl pyrazine	0.3±0.01	0.77±0.00	0.14±0.03	1±0.01	0.64±0.01	1.87±0.03	2.29±0.34	6.47±0.13	0.01±0.00	-
39	2,6-dimethyl pyrazine	1.06±0.01	0.6±0.04	-	0.18±0.00	1.47±0.03	0.57±0.03	-	0.93±0.08	-	0.01±0.00
40	2,3-dimethyl pyrazine	-	0.35±0.01	-	-	-	-	0.32±0.12	-	-	-
41	2-methyl-pyridine	-	0.05±0.00	-	-	-	0.02±0.00	-	-	-	-
42	2-ethyl-6-methyl pyrazine	0.64±0.03	0.25±0.01	-	0.2±0.00	-	0.3±0.01	-	-	-	-
43	2-ethyl-3-methyl pyrazine	-	0.23±0.00	0.14±0.02	0.14±0.01	-	0.3±0.01	0.39±0.04	0.82±0.1	-	-
44	2-ethyl-5-methyl pyrazine	0.26±0.02	0.03±0.00	0.06±0.01	0.16±0.00	0.32±0.02	0.32±0.02	0.78±0.07	0.84±0.02	-	-

45	2-ethyl pyrazine	-	0.54±0.01	-	-	-	0.96±0.04	-	-	-	-
46	1h-pyrrole-2-carboxaldehyde,1-ethyl-	0.19±0.02	0.14±0.01	-	-	-	0.06±0.00	-	-	-	-
47	trimethyl pyrazine	-	-	-	-	-	-	-	-	-	-
48	ethanone,1-(1h-pyrrol-2-yl)-	0.39±0.01	0.17±0.00	0.04±0.00	0.06±0.01	0.44±0.05	0.26±0.03	1.17±0.00	0.46±0.02	-	-
49	3-ethyl-2,5-dimethyl pyrazine	-	-	0.24±0.01	-	0.74±0.03	0.97±0.02	3.55±0.03	-	0.01±0.00	-
50	1-pyrrolidine carboxaldehyde	0.41±0.06	-	-	-	-	-	-	-	-	-
51	2-ethyl-3,5-dimethyl pyrazine	0.03±0.00	-	-	0.37±0.01	-	-	0.19±0.01	2.27±0.05	-	-
52	2-methyl-5-(2-propenyl) pyrazine,	-	-	-	-	-	-	0.07±0.01	-	-	-
53	(1-methylethenyl) pyrazine	0.08±0.00	0.03±0.00	-	-	-	-	-	-	-	-
54	3-pyridinol	0.28±0.05	0.3±0.01	-	-	-	-	-	-	-	-
55	1-(6-methyl-2-pyrazinyl)-1-ethanone	-	-	-	-	-	-	0.36±0.01	-	-	-
56	3-hydroxypyridine monoacetate	-	-	-	-	0.07±0.01	-	-	-	-	-
57	3-methoxy pyridine	-	0.13±0.01	-	-	-	-	-	-	-	-
58	isopropenyl pyrazine	-	0.02±0.00	-	-	-	-	-	-	-	-
59	1-(2-pyridinyl) ethanone	-	0.02±0.00	-	-	-	0.02±0.00	-	-	-	-
60	indolizine	0.02±0.00	-	-	-	0.04±0.00	-	-	-	-	-
61	1h-pyrrole-2-carboxaldehyde	1.24±0.41	2.31±0.00	-	0.13±0.01	-	1.01±0.02	0.5±0.12	0.54±0.01	-	-
62	1h-pyrrole-2-carboxaldehyde, 1-methyl-	-	0.3±0.01	-	-	-	0.1±0.00	-	0.2±0.01	-	-
63	2-pyrrolidinone	0.08±0.01	0.21±0.00	0.03±0.00	0.01±0.00	0.12±0.02	0.16±0.01	-	0.26±0.03	-	-
	**4 O-heterocycles**										
64	2-pentyl-furan	-	0.1±0.01	-	0.02±0.00	-	-	-	-	-	-
65	ethanone,1-(2-furanyl)-	-	0.85±0.05	-	0.27±0.02	-	0.8±0.01	-	1.41±0.13	-	-
66	3-(1-cyclopentenyl)furan	-	-	-	-	-	-	-	0.01±0.00	-	-
67	4h-pyran-4-one,2,3-dihydro-3,5-dihydroxy-6-methyl-	-	-	-	-	0.81±0.04	0.23±0.01	1.1±0.06	0.13±0.00	-	-
	**Alcohols**										

68	2-furanmethanol	0.47±0.08	1.43±0.00	0.24±0.02	0.2±0.02	0.35±0.03	0.36±0.01	-	-	-	-
69	1-hexanol	-	-	-	-	0.41±0.04	0.31±0.03	-	-	-	-
70	1,5-hexadien-3-ol	4.82±0.09	-	2.29±0.01	-	7.54±0.05	-	2.1±0.2	-	0.09±0.01	-
71	1-pentanol	-	-	-	-	-	-	-	-	-	0.02±0.00
72	(E)-2-octen-1-ol	-	-	-	-	-	-	-	-	-	-
73	1-octanol	-	-	-	0.04±0.00	-	-	-	-	-	-
74	phenylethyl alcohol	-	-	-	0.08±0.00	0.1±0.00	0.05±0.00	-	-	-	-
75	nicotinyl alcohol	-	0.14±0.01	-	-	-	0.1±0.01	-	-	-	-
76	benzyl alcohol	-	-	-	0.02±0.00	-	-	-	-	-	-
77	2h-1-benzopyran-3,4-diol,2-(3,4-dimethoxyphenyl)-3,4	1.35±0.05	0.18±0.02	0.26±0.00	-	2.84±0.68	-	5.77±1.08	0.87±0.15	0.03±0.00	-
78	maltol	0.12±0.01	-	-	-	0.13±0.02	-	0.6±0.01	-	-	-
79	4-ethyl-2-methoxy phenol	-	-	-	-	-	-	-	-	-	-
80	2-methoxy-4-vinylphenol	0.18±0.00	-	0.04±0.00	-	0.37±0.05	0.07±0.00	0.9±0.09	-	-	-
	**Aldehydes**										
81	acetaldehyde	0.14±0.01	0.37±0.02	-	-	0.28±0.01	0.36±0.02	-	0.42±0.02	-	0.08±0.00
82	(E)-2-butenal	-	-	-	0.64±0.01	-	-	1.16±0.13	2.06±0.13	-	-
83	(E)-2-pentenal	-	0.06±0.01	-	-	-	-	-	-	0.01±0.00	0.04±0.00
84	propanal	-	-	-	-	-	-	-	-	-	-
85	hexanal	0.27±0.01	0.45±0.03	0.05±0.00	0.91±0.02	0.29±0.04	0.47±0.03	2.7±0.44	5.04±0.14	0.09±0.00	0.38±0.00
86	2-methyl butanal,	-	-	-	-	-	0.12±0.01	-	0.14±0.00	-	-
87	3-methyl butanal,	-	-	-	-	-	0.3±0.00	-	-	-	-
88	furfural	1.66±0.13	4.78±0.02	1.38±0.06	2.97±0.01	6.19±0.21	12.83±0.03	11.2±0.26	16.09±0.15	-	0.1±0.01
89	(E)-2-hexenal	-	0.02±0.00	-	0.11±0.00	-	0.04±0.00	-	0.2±0.01	-	0.01±0.00
90	heptanal	-	-	0.02±0.00	0.24±0.00	-	-	-	-	0.05±0.00	0.06±0.00
91	(Z)-2-heptenal	-	-	-	-	-	-	9.62±0.55	-	0.08±0.00	-

92	5-methyl-2-furancarboxaldehyde	2.32±0.04	2.79±0.03	0.78±0.04	0.87±0.02	4.81±0.12	4.71±0.06	4.48±0.59	3.69±0.11	0.01±0.00	0.04±0.00
93	(E,E)-2,4-nonadienal	-	-	-	-	-	-	-	-	-	-
94	octanal	0.12±0.01	0.19±0.00	0.04±0.00	0.06±0.00	-	0.1±0.01	-	0.42±0.03	0.02±0.00	-
95	(E,E)-2,4-heptadienal	-	-	-	0.2±0.01	-	0.17±0.02	0.87±0.07	-	-	-
96	benzeneacetaldehyde	-	-	-	-	0.22±0.00	-	-	-	-	-
97	(E)-2-octenal	0.04±0.01	0.01±0.00	0.01±0.00	0.02±0.00	0.04±0.01	0.06±0.00	1.93±0.07	1.26±0.1	0.02±0.00	-
98	nonanal	0.34±0.02	0.17±0.00	0.11±0.01	0.21±0.00	0.55±0.02	0.21±0.01	1.39±0.08	-	0.05±0.00	0.1±0.00
99	benzaldehyde	-	0.19±0.01	-	0.19±0.00	-	0.26±0.04	-	0.56±0.02	-	-
100	(E,Z)-2,6-nonadienal	-	-	-	-	-	-	-	-	-	-
101	(E)-2-nonenal	-	-	-	-	0.04±0.00	-	0.73±0.06	-	-	-
102	4-ethyl-benzaldehyde	-	-	-	0.01±0.00	-	0.03±0.00	-	-	-	0.01±0.00
103	decanal	-	-	-	-	0.02±0.01	-	0.05±0.00	-	-	-
104	5-hydroxymethylfurfural	-	-	0.11±0.00	-	0.45±0.03	0.12±0.01	1.92±0.14	0.52±0.06	-	-
105	(E)-2-decenal	0.04±0.00	-	0.02±0.00	0.02±0.00	0.2±0.02	-	1.08±0.07	-	-	-
106	benzeneacetaldehyde,α-ethylidene-	0.08±0.01	-	-	-	-	-	-	-	-	-
107	2,4-decadienal	-	-	0.04±0.01	-	0.29±0.04	-	0.42±0.03	-	-	-
108	(E,E)-2,4-decadienal	-	-	-	-	0.06±0.01	-	1.04±0.07	-	-	-
109	2-undecenal	-	-	0.01±0.00	-	0.02±0.01	-	0.16±0.02	-	-	-
110	3-hydroxy-4-methoxy-benzaldehyde	-	-	-	-	0.01±0.00	-	-	-	-	-
	**Acids**										
111	formic acid	-	0.2±0.01	-	0.14±0.00	-	0.44±0.01	3.29±0.3	3.85±0.2	-	-
112	acetic acid	8.43±0.29	14.5±0.04	1.28±0.06	3.15±0.02	9.09±0.54	12.74±0.01	7.2±0.3	8.23±0.13	0.19±0.01	0.71±0.01
113	butanoic acid	-	-	-	-	-	-	-	-	0.01±0.00	0.06±0.00
114	4-pentenoic acid	0.16±0.01	1.02±0.01	0.02±0.00	0.32±0.02	0.02±0.00	0.67±0.01	0.18±0.01	2.15±0.1	-	-
115	pentanoic acid	-	0.06±0.00	0.02±0.00	0.04±0.00	-	-	-	-	-	0.01±0.00

116	hexanoic acid	-	0.36±0.01	0.03±0.00	0.17±0.02	-	0.29±0.03	-	1.57±0.17	-	0.1±0.01
117	heptanoic acid	-	-	-	0.1±0.00	0.07±0.01	-	-	-	0.01±0.00	-
118	benzoic acid	-	-	-	-	-	-	-	-	-	-
119	octanoic acid	-	0.4±0.02	0.09±0.01	0.23±0.00	0.11±0.02	-	-	-	0.02±0.00	0.04±0.00
120	crotonic acid	-	-	-	-	-	0.03±0.00	-	-	-	-
121	nonanoic acid	0.47±0.06	0.22±0.02	0.09±0.01	0.32±0.02	0.65±0.11	0.02±0.00	0.3±0.02	-	0.15±0.01	0.15±0.00
122	5-hexenoic acid	-	0.16±0.00	-	0.02±0.00	-	0.06±0.00	-	0.5±0.01	-	-
123	undecanoicacid	0.03±0.00	-	-	-	-	-	-	-	0.01±0.00	-
124	n-decanoicacid	-	-	-	0.03±0.00	0.01±0.00	-	-	-	-	-
125	propanoicacid	0.27±0.05	0.5±0.02	-	0.22±0.01	-	0.53±0.00	-	0.63±0.05	-	0.23±0.01
	**Ketones**										
126	2-decanone	-	-	-	-	-	-	-	-	0.01±0.00	-
127	4h-pyran-4-one,3,5-dihydroxy-2-methyl-	-	-	-	-	0.1±0.01	-	0.11±0.01	-	-	-
128	2-propanone,1-hydroxy-	-	0.62±0.02	-	0.32±0.01	1.36±0.15	1.12±0.01	-	-	-	-
129	3(2H)-furanone,dihydro-2-methyl	0.07±0.00	-	0.02±0.00	0.1±0.00	0.11±0.00	-	0.11±0.02	-	-	-
130	2,3-pentanedione	-	0.08±0.01	-	-	-	0.07±0.00	-	-	-	-
131	4-cyclopentene-1,3-dione	-	-	-	0.02±0.00	-	0.13±0.00	-	0.25±0.01	-	-
132	2-heptanone	0.03±0.00	-	-	0.09±0.01	-	-	-	-	0.02±0.00	0.03±0.00
133	3-penten-2-one	-	0.11±0.00	-	-	-	-	-	-	-	-
134	2(5H)-furanone	-	0.7±0.01	-	0.09±0.00	-	-	-	0.5±0.02	-	-
135	4-methyl-2-hexanone	-	0.06±0.00	-	-	-	-	-	0.13±0.01	-	-
136	isomaltol	0.04±0.01	-	-	-	0.03±0.00	-	-	-	-	-
137	5-hepten-2-one,6-methyl-	0.34±0.02	-	0.06±0.00	0.95±0.01	0.56±0.1	-	1.17±0.05	2.31±0.27	0.37±0.01	0.46±0.01
138	2-octanone	0.12±0.01	0.62±0.02	-	0.47±0.02	0.03±0.01	-	-	-	-	0.37±0.00
139	1,2-cyclopentanedione,3-methyl-	-	-	-	-	1.37±0.04	-	0.16±0.04	-	-	-

140	2-cyclopenten-1-one,2-hydroxy-3-methyl-	0.18±0.04	0.17±0.00	-	0.03±0.00	-	0.25±0.01	-	0.18±0.01	-	-
141	5-ethylfuran-2(5H)-one	-	-	-	-	-	-	-	-	-	-
142	3-octen-2-one	-	-	-	-	-	-	0.18±0.03	-	-	-
143	2,5-dimethylfuran-3,4(2h,5h)-dione	-	-	-	-	-	-	0.24±0.01	-	-	-
144	furaneol	-	0.07±0.01	-	-	0.09±0.01	0.06±0.00	-	-	-	-
145	(E,E)-3,5-octadien-2-one	0.03±0.00	-	-	-	0.03±0.00	-	-	-	-	-
146	3,5-octadien-2-one	-	-	-	-	-	-	0.03±0.00	-	-	-
147	acetophenone	-	-	-	0.01±0.00	-	-	-	-	-	-
148	ethanone,1-(2-hydroxy-5-methylphenyl)-	-	0.04±0.00	-	0.05±0.00	-	-	-	0.14±0.01	-	0.02±0.00
149	5-hepten-3-one, 5-methyl-	-	-	-	-	-	8.16±0.1	-	8.14±0.04	-	-
150	ethanone,1-(2,6-dihydroxy-4-methoxyphenyl)-	0.03±0.00	-	-	-	-	-	-	-	-	-
151	2(3H)-furanone,5-methyl-	-	0.04±0.00	-	-	-	0.04±0.00	-	-	-	-
152	2(3h)-furanone,dihydro-3-hydroxy-4,4-dimethyl-,(±)-	-	-	-	-	0.15±0.02	-	-	-	-	-
	**olefins**										
153	2-methyl-1,5-heptadiene(c,t)	-	-	0.04±0.00	-	-	-	0.59±0.01	0.68±0.07	0.01±0.00	-
154	γ-terpinene	-	-	-	-	-	0.07±0.00	-	-	-	-
	**esters**										
155	1-propen-2-ol, acetate	-	-	-	-	-	0.36±0.01	-	0.59±0.07	-	-
156	butyrolactone	-	-	-	0.25±0.00	-	0.58±0.00	-	-	-	-
157	2(3H)-furanone,dihydro-5-pentyl-	0.01±0.00	-	-	0.04±0.00	0.02±0.00	-	0.06±0.00	-	-	-
158	propanoic acid, 2-propenyl ester	-	3.22±0.00	-	2.23±0.04	-	-	-	-	-	-

“-”: not detected.

Table S2 Volatile compounds and their contents in fragrant rapeseed oils

NO.	Name	S6 (HP-5MS)	S6 (DB-WAX)	S7 (HP-5MS)	S7 (DB-WAX)	S8 (HP-5MS)	S8 (DB-WAX)	S9 (HP-5MS)	S9 (DB-WAX)
	**Nitriles**								
1	3-butenenitrile	0.28±0.04	1.09±0.03	-	0.34±0.00	-	0.14±0.00	-	-
2	methylacrylonitrile	-	-	-	0.18±0.00	-	-	-	0.23±0.01
3	2-butenenitrile	0.57±0.00	1.07±0.01	0.1±0.00	0.36±0.00	-	0.04±0.00	-	0.32±0.00
4	2,4-pentadienenitrile	0.42±0.03	0.94±0.03	0.37±0.05	0.88±0.01	0.03±0.00	0.22±0.00	0.07±0.01	0.22±0.00
5	(ch3)2c=chcn	46.43±4	52.36±1.7	4.8±0.1	16.14±0.19	2.19±0.03	9.77±0.09	5.51±0.03	16.25±0.05
6	5-cyano-1-pentene	22.06±3.16	23.94±0.14	2.03±0.11	5.28±0.04	1.77±0.02	5.19±0.06	3.86±0.06	9.01±0.14
7	2-methyl-butanenitrile	-	0.25±0.01	-	-	-	-	-	-
8	5-methyl-hexanenitrile	0.47±0.08	-	-	-	-	0.22±0.00	-	0.33±0.03
9	isoamyl cyanide	-	-	-	-	-	-	-	-
10	heptanonitrile	-	0.98±0.12	-	0.27±0.00	-	0.07±0.01	-	0.37±0.02
11	3-pentenenitrile	-	1.45±0.15	-	-	-	-	-	-
12	2-pentenenitrile	-	0.04±0.00	-	-	-	0.02±0.00	-	-
13	hexanenitrile	-	0.41±0.01	-	-	-	0.09±0.00	-	0.33±0.00
14	benzylnitrile	-	0.22±0.02	-	-	-	-	-	0.08±0.01
15	benzenepropanenitrile	30.76±2.96	7.05±0.03	1.18±0.07	0.43±0.03	1.51±0.1	1.16±0.09	4.98±0.33	2.02±0.2
	**Sulfides**								
16	thiazole	-	-	-	0.06±0.00	-	-	-	-
17	5-thiazoleethanol,4-methyl-	-	0.03±0.00	-	-	-	-	-	-
18	carbon disulfide	5.42±0.18	4.41±0.13	2.32±0.15	2.99±0.08	0.07±0.02	0.13±0.00	0.34±0.02	0.79±0.04
19	dimethyl disulfide	0.04±0.00	0.06±0.00	-	0.09±0.00	0.04±0.00	0.16±0.00	-	0.03±0.00
20	dimethyl sulfoxide	-	1.09±0.05	-	0.57±0.02	0.01±0.00	0.06±0.00	0.01±0.00	0.14±0.01
21	2(3h)-thiophenone,dihydro-5-methyl-	1.69±0.3	2.15±0.06	0.26±0.00	-	-	-	0.14±0.01	-

22	dimethyl trisulfide	-	0.01±0.00	-	-	-	0.22±0.00	-	0.07±0.00
23	cyano-3,4-epithiobutane	0.41±0.09	-	-	-	-	-	-	-
24	5-ethyl-4-methylthiazole	0.07±0.01	-	-	-	-	-	-	-
25	pentanenitrile,5-(methylthio)-	1.61±0.18	-	-	-	-	-	-	-
26	6-(methylthio)hexanenitrile	2.08±0.11	-	-	-	-	-	-	-
27	methanethiol	-	0.04±0.00	-	0.12±0.00	-	-	-	-
28	2-thiophenemethanol	0.38±0.01	0.23±0.01	-	-	-	-	-	0.02±0.00
29	3-thiophenecarboxaldehyde	-	0.67±0.03	-	0.18±0.01	-	-	-	-
30	butane,2-isothiocyanato	0.19±0.03	-	-	-	0.02±0.00	-	0.07±0.00	-
31	1-butene,4-isothiocyanato	11.36±1.59	10.4±0.12	0.82±0.05	0.59±0.06	0.63±0.04	0.66±0.01	2.52±0.08	2.11±0.12
32	benzene,(2-isothiocyanatoethyl)-	0.26±0.02	-	-	-	-	-	-	-
	**N-heterocycles**								
33	1,3-diazine	-	0.09±0.01	-	0.14±0.00	-	-	-	-
34	pyrazine	-	-	0.05±0.00	-	-	-	-	-
35	pyridine	0.21±0.01	0.26±0.03	0.1±0.03	0.73±0.01	-	0.2±0.00	0.01±0.00	-
36	3-methyl pyridine	0.09±0.01	-	0.03±0.01	-	-	-	-	-
37	methyl pyrazine	1.53±0.14	-	0.62±0.05	-	0.1±0.00	-	0.11±0.01	-
38	2,5-dimethyl pyrazine	2.41±0.33	3.57±0.02	0.11±0.01	0.78±0.05	0.3±0.01	0.77±0.02	0.3±0.00	1.23±0.01
39	2,6-dimethyl pyrazine	-	1.47±0.02	0.51±0.11	0.48±0.05	0.17±0.02	0.11±0.00	0.57±0.01	0.18±0.00
40	2,3-dimethyl pyrazine	0.3±0.06	-	-	0.4±0.00	-	-	-	-
41	2-methyl-pyridine	-	-	-	-	-	-	-	-
42	2-ethyl-6-methyl pyrazine	-	-	-	0.16±0.02	-	0.09±0.01	-	0.27±0.00
43	2-ethyl-3-methyl pyrazine	1.24±0.01	0.95±0.18	-	0.15±0.00	-	0.12±0.00	-	-
44	2-ethyl-5-methyl pyrazine	1.34±0.14	0.87±0.06	0.14±0.00	-	0.06±0.02	0.13±0.00	-	0.15±0.00
45	2-ethyl pyrazine	-	-	-	0.74±0.03	-	-	-	-
46	1h-pyrrole-2-carboxaldehyde,1-ethyl-	-	0.12±0.01	0.13±0.01	0.09±0.00	-	-	-	-

47	trimethyl pyrazine	-	-	-	0.14±0.00	-	-	-	-
48	ethanone,1-(1h-pyrrol-2-yl)-	1.9±0.13	0.65±0.03	0.37±0.02	0.12±0.01	0.03±0.01	0.03±0.00	0.28±0.04	0.06±0.00
49	3-ethyl-2,5-dimethyl pyrazine	4±0.58	1.75±0.11	0.45±0.05	-	0.33±0.02	-	0.66±0.01	-
50	1-pyrrolidine carboxaldehyde	-	-	-	-	-	-	-	-
51	2-ethyl-3,5-dimethyl pyrazine	-	-	0.01±0.00	-	-	0.48±0.00	-	0.57±0.04
52	2-methyl-5-(2-propenyl) pyrazine,	0.08±0.00	-	-	-	-	-	-	-
53	(1-methylethenyl) pyrazine	0.12±0.01	-	0.09±0.02	0.01±0.00	-	-	-	-
54	3-pyridinol	-	0.15±0.02	0.12±0.03	0.19±0.02	-	-	-	-
55	1-(6-methyl-2-pyrazinyl)-1-ethanone	0.38±0.02	-	-	-	0.01±0.00	-	-	-
56	3-hydroxypyridine monoacetate	-	-	0.09±0.01	-	-	-	-	-
57	3-methoxy pyridine	-	-	-	0.15±0.01	-	-	-	-
58	isopropenyl pyrazine	-	0.03±0.00	-	-	-	-	-	-
59	1-(2-pyridinyl) ethanone	-	0.04±0.00	-	0.01±0.00	-	-	-	-
60	indolizine	0.11±0.02	-	-	-	-	-	-	-
61	1h-pyrrole-2-carboxaldehyde	2.72±0.49	2.46±0.15	3.81±0.54	1.87±0.03	-	0.02±0.00	-	0.06±0.00
62	1h-pyrrole-2-carboxaldehyde, 1-methyl-	-	0.39±0.04	-	0.23±0.00	-	-	-	-
63	2-pyrrolidinone	-	0.41±0.02	0.16±0.01	0.16±0.01	0.04±0.00	0.13±0.00	-	-
	**4 O-heterocycles**								
64	2-pentyl-furan	-	-	-	-	-	-	-	-
65	ethanone,1-(2-furanyl)-	-	2.11±0.07	-	0.62±0.04	-	0.08±0.01	-	0.18±0.01
66	3-(1-cyclopentenyl)furan	-	-	-	0.02±0.00	-	-	-	-
67	4h-pyran-4-one,2,3-dihydro-3,5-dihydroxy-6-methyl-	1.39±0.22	0.08±0.01	-	-	-	-	-	-
	**Alcohols**								
68	2-furanmethanol	4.55±0.01	0.57±0.01	0.04±0.00	0.3±0.00	0.03±0.00	0.01±0.00	0.06±0.01	0.04±0.00
69	1-hexanol	-	-	-	-	-	-	-	-
70	1,5-hexadien-3-ol	18.93±2	-	5.8±0.1	3.18±0.01	3.05±0.05	-	2.58±0.16	-

71	1-pentanol	-	-	-	-	-	0.02±0.00	-	-
72	(E)-2-octen-1-ol	-	-	-	-	0.01±0.00	-	0.03±0.00	-
73	1-octanol	-	-	-	-	-	0.02±0.00	-	-
74	phenylethyl alcohol	-	-	-	-	-	0.02±0.00	-	0.04±0.00
75	nicotinyl alcohol	1.71±0.16	-	-	-	-	-	-	0.02±0.00
76	benzyl alcohol	-	-	-	-	-	-	-	-
77	2h-1-benzopyran-3,4-diol,2-(3,4-dimethoxyphenyl)-3,4	-	0.54±0.04	-	0.06±0.01	1.02±0.05	0.16±0.01	2.35±0.24	-
78	maltol	1.64±0.18	0.13±0.01	0.04±0.00	-	-	-	-	-
79	4-ethyl-2-methoxy phenol	-	-	-	-	-	-	0.37±0.05	0.08±0.01
80	2-methoxy-4-vinylphenol	1.93±0.08	-	0.16±0.02	0.02±0.00	0.08±0.01	0.03±0.00	-	0.06±0.00
	**Aldehydes**								
81	acetaldehyde	0.33±0.00	0.32±0.03	0.41±0.00	0.46±0.01	-	0.08±0.01	-	-
82	(E)-2-butenal	-	0.46±0.01	-	0.63±0.02	0.02±0.00	-	-	0.9±0.01
83	(E)-2-pentenal	-	-	-	0.09±0.00	-	-	0.21±0.02	0.6±0.01
84	propanal	-	-	-	-	-	-	-	0.58±0.04
85	hexanal	0.38±0.07	-	0.22±0.01	0.59±0.04	0.12±0.01	0.52±0.00	0.76±0.05	2.24±0.00
86	2-methyl butanal,	-	0.15±0.01	-	0.13±0.01	-	-	-	-
87	3-methyl butanal,	-	0.34±0.05	-	0.29±0.02	-	-	-	-
88	furfural	19.53±1.61	23.86±0.14	3.69±0.68	6.21±0.11	0.23±0.01	0.87±0.02	1±0.11	1.84±0.12
89	(E)-2-hexenal	-	0.05±0.00	-	0.03±0.00	0.01±0.00	0.04±0.00	0.06±0.01	0.32±0.02
90	heptanal	-	-	0.11±0.02	-	0.05±0.00	0.16±0.00	-	0.58±0.02
91	(Z)-2-heptenal	-	-	0.46±0.02	-	0.13±0.02	-	-	-
92	5-methyl-2-furancarboxaldehyde	16.12±2.04	11.03±0.37	3.56±0.03	2.72±0.04	0.22±0.01	0.19±0.01	0.48±0.01	0.66±0.01
93	(E,E)-2,4-nonadienal	0.12±0.00	-	-	-	-	-	-	0.07±0.00
94	octanal	-	-	-	0.12±0.01	0.06±0.02	0.03±0.00	-	0.19±0.02
95	(E,E)-2,4-heptadienal	-	0.27±0.04	-	-	-	0.11±0.01	2.49±0.36	1.26±0.04

96	benzeneacetaldehyde	0.28±0.07	-	-	-	-	-	-	-
97	(E)-2-octenal	-	0.13±0.01	0.05±0.01	0.01±0.00	0.01±0.00	0.01±0.00	0.4±0.06	0.22±0.01
98	nonanal	1.21±0.26	-	0.42±0.01	0.11±0.00	0.01±0.00	0.08±0.00	1.07±0.02	0.62±0.01
99	benzaldehyde	-	0.45±0.09	-	0.14±0.01	-	0.07±0.01	-	0.41±0.01
100	(E,Z)-2,6-nonadienal	-	-	-	-	-	-	0.16±0.00	0.08±0.00
101	(E)-2-nonenal	-	-	0.02±0.00	-	0.02±0.00	-	1.23±0.03	0.42±0.00
102	4-ethyl-benzaldehyde	-	-	-	-	-	0.01±0.00	-	0.05±0.00
103	decanal	-	-	-	-	-	-	-	-
104	5-hydroxymethylfurfural	6.33±0.38	0.81±0.1	-	-	0.01±0.00	-	0.12±0.01	-
105	(E)-2-decenal	0.97±0.09	-	0.09±0.01	0.01±0.00	0.02±0.00	0.09±0.01	0.29±0.02	0.08±0.00
106	benzeneacetaldehyde,α-ethylidene-	-	-	0.04±0.01	-	-	-	-	-
107	2,4-decadienal	-	-	0.08±0.01	-	0.02±0.00	-	0.74±0.07	0.28±0.02
108	(E,E)-2,4-decadienal	-	-	-	-	0.06±0.01	-	-	0.08±0.00
109	2-undecenal	0.29±0.03	-	0.03±0.00	-	-	-	0.13±0.01	0.08±0.01
110	3-hydroxy-4-methoxy-benzaldehyde	0.07±0.01	-	-	-	-	-	-	-
	**Acids**								
111	formic acid	-	1.07±0.06	0.77±0.11	0.12±0.01	-	-	1.32±0.03	1.12±0.09
112	acetic acid	23.96±1.84	19.9±0.19	10.44±0.34	14.76±0.08	0.55±0.03	1.15±0.01	0.96±0.02	3.23±0.06
113	butanoic acid	-	-	-	-	-	0.11±0.00	0.06±0.01	0.59±0.01
114	4-pentenoic acid	0.12±0.01	2.66±0.04	-	0.88±0.03	0.03±0.00	0.13±0.00	-	0.35±0.01
115	pentanoic acid	-	-	-	-	0.03±0.00	0.04±0.00	0.01±0.00	0.32±0.04
116	hexanoic acid	-	0.62±0.03	-	0.24±0.01	0.04±0.00	0.16±0.02	-	0.71±0.00
117	heptanoic acid	-	-	0.07±0.00	0.06±0.01	-	-	-	0.19±0.00
118	benzoic acid	0.17±0.00	0.1±0.02	-	-	-	-	-	-
119	octanoic acid	-	-	0.18±0.00	-	0.07±0.00	0.11±0.01	0.47±0.02	0.32±0.03
120	crotonic acid	-	0.07±0.01	-	-	-	-	-	-

121	nonanoic acid	-	-	0.87±0.02	0.26±0.02	0.23±0.03	0.15±0.00	0.89±0.1	0.56±0.04
122	5-hexenoic acid	-	0.54±0.05	-	0.12±0.00	-	0.02±0.00	-	0.09±0.00
123	undecanoicacid	-	-	-	-	-	-	0.05±0.00	-
124	n-decanoicacid	-	-	-	-	0.03±0.00	-	-	-
125	propanoicacid	-	0.85±0.05	0.22±0.01	0.56±0.01	-	0.2±0.00	0.24±0.03	0.6±0.01
	**Ketones**								
126	2-decanone	-	-	-	-	0.02±0.00	-	-	-
127	4h-pyran-4-one,3,5-dihydroxy-2-methyl-	1.89±0.13	-	-	-	-	-	-	-
128	2-propanone,1-hydroxy-	-	-	0.99±0.19	0.87±0.01	-	-	-	0.18±0.00
129	3(2H)-furanone,dihydro-2-methyl	0.37±0.05	-	0.05±0.01	-	-	-	-	-
130	2,3-pentanedione	-	-	-	0.04±0.00	-	-	-	-
131	4-cyclopentene-1,3-dione	0.32±0.01	0.43±0.05	-	-	-	-	-	-
132	2-heptanone	-	-	-	-	-	0.03±0.00	0.08±0.01	0.17±0.01
133	3-penten-2-one	-	0.07±0.01	-	0.12±0.01	-	-	-	-
134	2(5H)-furanone	-	0.88±0.01	-	0.62±0.00	-	0.03±0.00	-	-
135	4-methyl-2-hexanone	-	0.08±0.01	-	-	-	-	-	-
136	isomaltol	0.12±0.01	-	-	-	-	-	-	-
137	5-hepten-2-one,6-methyl-	-	1.49±0.00	0.76±0.02	-	0.31±0.00	0.8±0.02	1.79±0.06	2.36±0.02
138	2-octanone	-	-	0.15±0.01	0.65±0.01	-	0.28±0.01	-	0.57±0.07
139	1,2-cyclopentanedione,3-methyl-	1.02±0.17	-	0.26±0.02	-	-	-	-	-
140	2-cyclopenten-1-one,2-hydroxy-3-methyl-	-	0.54±0.03	-	0.11±0.01	-	-	-	-
141	5-ethylfuran-2(5H)-one	-	-	-	-	0.01±0.00	-	0.06±0.01	0.07±0.00
142	3-octen-2-one	-	-	-	-	-	-	0.04±0.01	-
143	2,5-dimethylfuran-3,4(2h,5h)-dione	-	-	-	-	-	-	-	-
144	furaneol	-	-	-	0.03±0.00	-	-	-	-
145	(E,E)-3,5-octadien-2-one	-	-	0.04±0.01	-	0.01±0.00	-	0.49±0.01	0.16±0.01

146	3,5-octadien-2-one	-	-	-	-	0.01±0.00	-	0.48±0.09	-
147	acetophenone	-	-	-	-	-	-	-	0.02±0.00
148	ethanone,1-(2-hydroxy-5-methylphenyl)-	-	-	-	-	-	-	0.32±0.04	-
149	5-hepten-3-one, 5-methyl-	-	7.01±0.13	-	-	-	-	-	-
150	ethanone,1-(2,6-dihydroxy-4-methoxyphenyl)-	-	-	-	-	0.01±0.00	-	-	-
151	2(3H)-furanone,5-methyl-	-	0.09±0.00	-	0.04±0.00	-	-	-	-
152	2(3h)-furanone,dihydro-3-hydroxy-4,4-dimethyl-,(±)-	0.83±0.11	-	-	-	-	-	-	-
	**olefins**								
153	2-methyl-1,5-heptadiene(c,t)	-	-	0.1±0.01	-	-	-	-	-
154	γ-terpinene	-	0.22±0.01	-	-	-	-	-	-
	**esters**								
155	1-propen-2-ol, acetate	-	0.49±0.09	-	-	-	-	-	-
156	butyrolactone	-	-	-	0.6±0.00	-	0.13±0.00	-	0.29±0.00
157	2(3H)-furanone,dihydro-5-pentyl-	-	-	0.02±0.00	-	-	-	0.05±0.00	-
158	propanoic acid, 2-propenyl ester	-	-	-	-	-	2.56±0.11	-	3.47±0.04

“-”: not detected.

Table S3 Odor thresholds and Relative odor activity value analysis (ROAV＞0.1) of aroma compounds

No.[1]	Compounds	Odor threshold (mg/kg)[2]	Media	Relative odor activity value								
				S1	S2	S3	S4	S5	S6	S7	S8	S9
V1	methanethiol	0.00006	sunflower oil	100	-	51.288	-	-	49.823	92.917	-	-
V2	acetaldehyde	0.00022	sunflower oil	71.923	-	100	100	100	100	100	100	-
V3	propanal	0.0094	sunflower oil	-	-	-	-	-	-	-	-	45.622
V4	2-methyl butanal	0.0022	sunflower oil	-	-	3.481	3.407	-	4.751	2.758	-	-
V5	2,3-pentanedione	0.0003	sunflower oil	11.104	-	15.088	-	-	-	6.373	-	-
V6	dimethyl disulfide	0.012	deodorised edible oil	0.273	5.433	0.284	0.471	0.41	0.356	0.369	3.538	1.798
V7	2-heptanone	1.5	vegetable oil	-	0.107	-	-	0.006	-	-	0.006	0.083
V8	heptanal	0.05	sunflower oil	-	8.472	-	-	0.358	-	-	0.856	8.652
V9	2,5-dimethyl pyrazine	2	sunflower oil	0.016	0.894	0.058	0.168	-	0.121	0.019	0.104	0.456
V10	2,6-dimethyl pyrazine	0.13	rapeseed oil	0.199	2.512	0.273	0.373	0.021	0.768	0.176	0.233	1.004
V12	5-hepten-2-one, 6-methyl	1	deodorised olive oil	-	1.696	-	0.12	0.134	0.101	-	0.217	1.75
V13	dimethyl trisulfide	0.0042	sunflower oil	-	15.542	0.616	0.031	-	0.137	-	14.5	11.656
V14	2-ethyl-3-methyl pyrazine	0.5	water	0.02	0.513	0.037	0.085	-	0.129	0.014	0.068	-
V15	2-ethyl-6-methyl pyrazine	0.051	sunflower oil	0.209	6.985	0.359	-	-	-	0.154	0.481	3.89
V16	2-ethyl-5-methyl pyrazine	0.32	vegetable oil	0.004	0.902	0.061	0.137	-	0.185	-	0.111	0.352
V17	2-ethyl-3,5-dimethyl pyrazine	0.0075	sunflower oil	-	88.807	-	15.749	-	-	-	17.248	56.695
V18	1-butene, 4-isothiocyanato	0.07	rapeseed oil	0.456	24.584	2.119	6.94	-	10.095	0.402	2.567	22.283
V19	furfural	0.7	sunflower oil	0.292	7.551	1.13	1.194	0.041	2.315	0.422	0.338	1.949
V20	acetic acid	0.75	vegetable oil	0.827	7.481	1.047	0.57	0.273	1.802	0.936	0.415	3.19
V21	(*E*,*E*)-2,4-heptadienal	0.05	sunflower oil	-	6.967	0.206	-	-	0.362	-	0.586	18.633
V22	benzaldehyde	0.69	rapeseed oil	0.011	0.486	0.024	0.042	-	0.045	0.01	0.029	0.442
V23	(*E*)-2-nonenal	0.15	deodorised edible oil	-	-	-	-	-	-	-	-	2.089

V24	dimethyl sulfoxide	0.1	rapeseed oil	0.211	3.923	0.532	0.687	-	0.741	0.273	0.17	1.05
V25	5-methyl-2-furancarboxaldehyde	0.26	rapeseed oil	0.46	5.942	1.116	0.738	0.042	2.883	0.498	0.196	1.877
V26	(*E*,*Z*)-2,6-nonadienal	0.0038	sunflower oil	-	-	-	-	-	-	-	-	15.377
V27	Butanoic acid	0.205	vegetable oil	-	-	-	-	0.083	-	-	0.142	2.149
V29	2-furanmethanol	0.68	rapeseed oil	0.09	0.513	0.033	-	-	0.057	0.021	0.005	0.042
V30	(*E*,*E*)-2,4-nonadienal	0.0015	sunflower oil	-	-	-	-	-	-	-	-	33.042
V31	2(5H)-furanone	0.12	rapeseed oil	0.248	1.314	-	0.217	-	0.497	0.246	0.063	-
V32	2,4-decadienal	0.18	sunflower oil	-	-	-	-	-	-	-	-	0.315
V34	hexanoic acid	0.7	deodorised olive oil	0.022	0.443	0.025	0.116	0.04	0.06	0.016	0.061	0.754
V35	benzyl nitrile	0.07	rapeseed oil	-	0.571	0.027	0.129	-	0.211	-	-	0.829
V36	heptanoic acid	0.1	deodorised olive oil	-	1.795	-	-	-	-	0.029	-	1.385
V37	4-ethyl-2-methoxy phenol	0.05	sunflower oil	-	-	-	-	-	-	-	-	1.203
V38	benzenepropanenitrile	0.015	water	1.704	100	8.046	21.959	3.317	31.939	1.358	21.011	100

"-" Not detected

In each sample, the component that contributed the most to the overall flavor had an ROAV of 100.000.

[1] The numbers of compounds are consistent with their numbers in Table 1.

[2] Odor threshold according to Van Gemert (2015).

Table S4 Nine fragrant rapeseed oils information

Regions	Sample	Processing technology	Ingredients	Date of production
Sichuan province	S-1	Press	Rapeseed oil	29-Aug-19
Sichuan province	S-4	Press	Rapeseed oil	29-Jul-19
Sichuan Province	S-6	Press	Rapeseed oil	13-Aug-19
Sichuan province	S-9	Press	Rapeseed oil	2-Jul-19
Shanghai	S-2	Press	Rapeseed oil	1-Aug-19
Shanghai	S-3	Press	Rapeseed oil	5-Aug-19
Yunnan Province	S-5	Press	Rapeseed oil	10-Aug-19
Yunnan Province	S-8	Press	Rapeseed oil	17-Aug-19
Jiangsu Province	S-7	Press	Rapeseed oil	15-Jul-19

Table S5 Definition of sensory characteristics of fragrant rapeseed oils

Characteristic	Definition	Reference
**Roasted**	A flavor reminiscent of roasted nuts, such as roasted peanuts and roasted sesame	Roasted nuts: Fry walnuts over low heat for 10 minutes
**Pickle-like**	A flavor reminiscent of pickled vegetables, which is slightly salty and has the flavor of lactic acid	Pickles: Lactic acid fermented Chinese cabbage
**Burnt**	A flavor reminiscent of burnt grains or oil crops	Burnted nuts: 800W microwave walnuts for 5 minutes
**Green**	A flavor reminiscent of raw rapeseeds or sprouted beans	Raw rapeseed: Fresh rapeseed
**Pungent**	A flavor reminiscent of pungent pepper	Olive oil: Extra virgin olive oil
**Puffed food-like**	A flavor reminiscent of salty and umami snack food	Rice crispies with senbei flavor: Want Want senbei rice crackers

Fig. S1 Traditional processing of fragrant rapeseed oil production

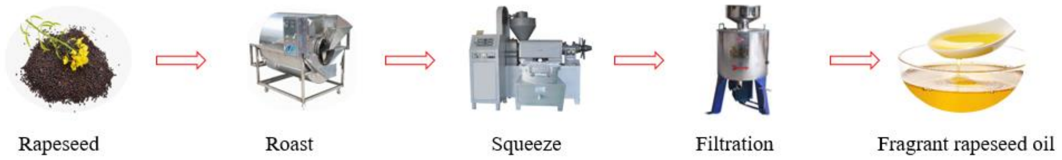

Fig. S2 Correlation analysis diagrams of sensory characteristics of fragrant rapeseed oils

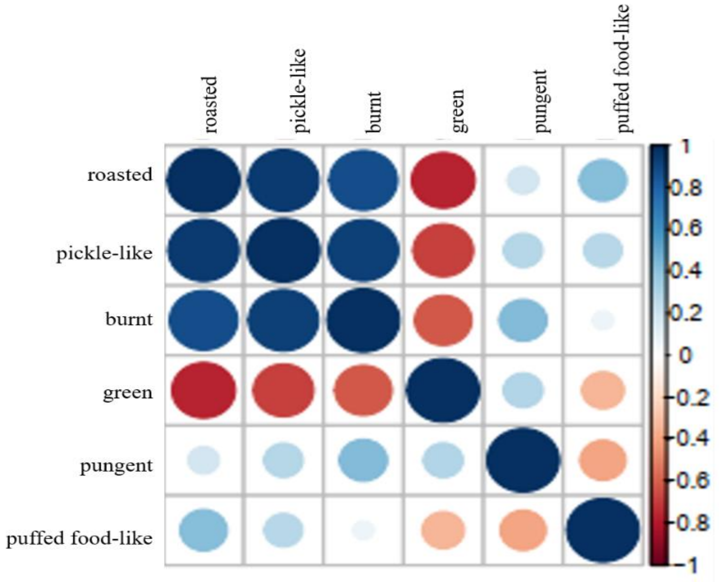

Fig. S3 Aroma compounds identification of 32 authentic standard compounds

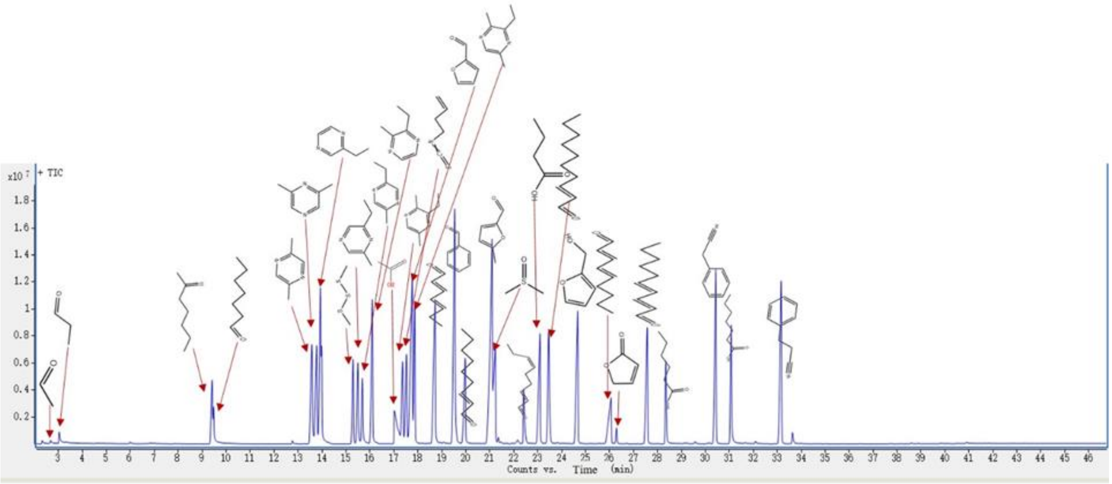

Fig. S4 Heat map results of 10 aroma compounds with a significant difference in fragrant rapeseed oil ($p < 0.05$).

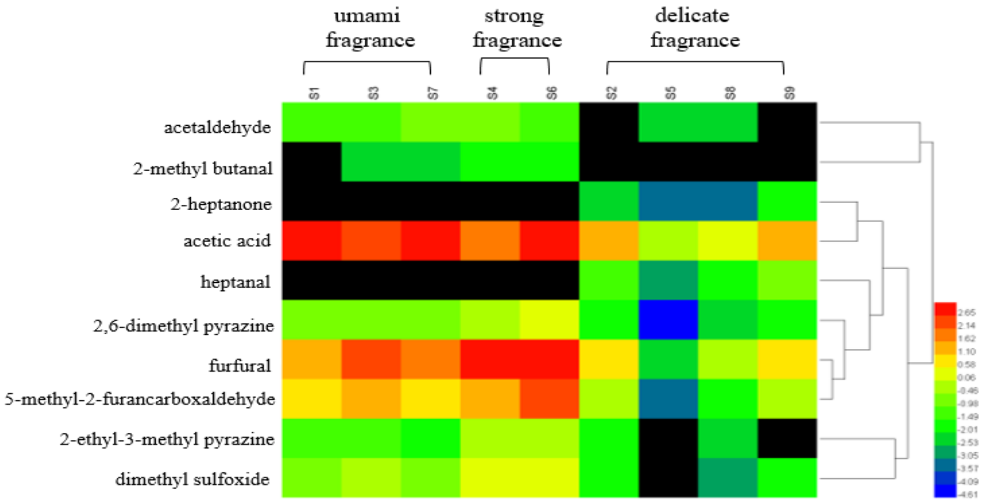

Supplement: Supplementary file 1 [file Data_Sheet_1.PDF]
